# Double-Sided Tape in Microfluidics: A Cost-Effective Method in Device Fabrication

**DOI:** 10.3390/bios14050249

**Published:** 2024-05-15

**Authors:** Savanah Smith, Marzhan Sypabekova, Seunghyun Kim

**Affiliations:** Department of Electrical & Computer Engineering, Baylor University, Waco, TX 76798, USA; savanah_smith1@baylor.edu (S.S.); marzhan_sypabekova@baylor.edu (M.S.)

**Keywords:** double-sided tape, microfluidic channel, rapid fabrication, laser cutting, xurography

## Abstract

The demand for easy-to-use, affordable, accessible, and reliable technology is increasing in biological, chemical, and medical research. Microfluidic devices have the potential to meet these standards by offering cost-effective, highly sensitive, and highly specific diagnostic tests with rapid performance and minimal sample volumes. Traditional microfluidic device fabrication methods, such as photolithography and soft lithography, are time-consuming and require specialized equipment and expertise, making them costly and less accessible to researchers and clinicians and limiting the applicability and potential of microfluidic devices. To address this, researchers have turned to using new low-cost materials, such as double-sided tape for microfluidic device fabrication, which offers simple and low-cost processes. The innovation of low-cost and easy-to-make microfluidic devices improves the potential for more devices to be transitioned from laboratories to commercialized products found in stores, offices, and homes. This review serves as a comprehensive summary of the growing interest in and use of double-sided tape-based microfluidic devices in the last 20 years. It discusses the advantages of using double-sided tape, the fabrication techniques used to create and bond microfluidic devices, and the limitations of this approach in certain applications.

## 1. Advancements in Microfluidics: Revolutionizing Diagnostics

Microfluidics is a multidisciplinary field representing the science and technology of controlling and manipulating small volumes of fluid in micrometer-sized channels [1,2,3]. Microfluidic devices consist of a single or network of microfluidic channels along with holes for fluid to enter and exit the device. The concept of microfluidics can be traced back to the 1950s–1960s, when researchers began to investigate the behavior of fluids at the microscale based on capillary flow concepts. These concepts were first used in inkjet printing allowing ink to travel through small channels and exit through a nozzle to form discrete drops [4,5]. The simple technology of fluid flow through a single channel led to the development of two of the most used microfluidic devices: glucose monitors and pregnancy tests. Over time, more components have been added to microfluidic devices to enable a more complex control and function. This led to the development of microfluidic devices as a field of research in the late 1990s. This research has expanded the applications of microfluidic devices towards use across many fields including medical diagnostics, drug discovery, and environmental monitoring. The outbreak of COVID-19 was the perfect opportunity for the rise of low-cost and simple diagnostic tools through microfluidic devices. The large demand and eventual success of on-site rapid testing led to an increased interest in the commercialization of microfluidic devices [6,7,8]. As of 2021, the market for microfluidic devices was estimated to be around USD 20 billion and is projected to greatly increase over the next decade as more applications are discovered and researched [9,10]. This is because the ability for the early and rapid detection of diseases is imperative to improving healthcare worldwide to provide life-saving diagnoses and treatments in a more timely manner [11,12,13].

**Figure 1:**
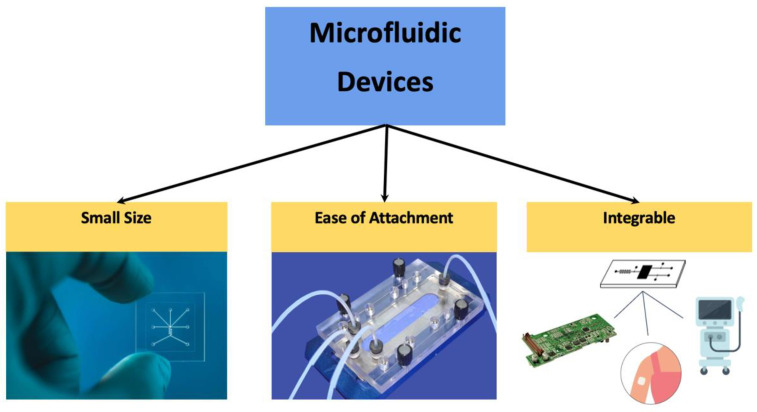
Microfluidic devices aid diagnostics and testing due to their small size and ability to connect to a variety of intermediate attachments. They are also readily integrated into complex systems. Left image: reproduced courtesy of Workshop of Photonics (WOP). Middle image: reproduced with permission from Microfluidic Chip Shop GmbH (Jena, 07747, Germany).

The complexity and advancement of microfluidic devices can occur through the addition of other systems and structures inside or outside of the microfluidic channels. Components like valves and pumps permit greater control over the flow of small volumes of fluids entering and exiting the channels. Microfluidic valves regulate fluidic paths for the allowance or restriction of flow across particular channels. Valves can be active or passive depending on the mechanism that controls the opening and closing of the valve [14]. Microfluidic pumps can also provide enhanced control through the pushing or pulling of fluid through a microfluidic channel. There are both mechanical and non-mechanical pumps based on the driving force. Common pumps include diaphragm, syringe, piezoelectric, and peristaltic [15]. These components can be easily attached externally to the microfluidic channel through inlet and outlet ports or fully integrated into the microfluidic chips to direct, mix, separate, or trap fluids and molecules [16,17]. Microfluidic devices can be readily integrated with other technologies such as printed circuit boards (PCBs), wearable technologies, or other systems for various applications. Figure 1 summarizes some of the benefits of microfluidic devices.

At the core of microfluidic devices are the microfluidic channels. Due to the small nature of their size, microfabrication methods are used for the buildup, definition, and sealing of microfluidic channels. Numerous fabrication techniques have been developed using chemical, mechanical, or other processes depending on the materials, complexity, and dimensions required. These methods typically require specific manufacturing methods and training which can be time, labor, and cost-intensive [1,18]. Recently, researchers have looked at new methods and materials for reducing fabrication time and cost. One of these low-cost materials is double-sided tape. Double-sided tape is advantageous for defining, bonding, and sealing microfluidic channels without complex or expensive processes. In this review, we attempt to summarize the benefits and use of double-sided tape for microfluidic channels within a wide range of microfluidic device applications.

## 2. Microfluidic Device Fabrication

### 2.1. Material Selection: Properties, Techniques, and Applications

Material choice is the first step when determining the fabrication process for a microfluidic channel. The choice of material depends on several factors: the desired properties of the device, available fabrication processes, and intended application.

Conventional fabrication methods such as photolithography and etching originated with the use of silicon substrates. Eventually, glass became a common substitute for silicon, given its optical transparency and greater biocompatibility, allowing for the development of optical-based detection for microfluidics [1,4,19]. Today, polymers or elastomeric materials such as polydimethylsiloxane (PDMS), polymethyl methacrylate (PMMA), or polycarbonate (PC) have become the most widely used material for microfluidics since their introduction in the late 1990s. These materials are less expensive and more flexible while maintaining optical transparency and the ability to create high-resolution three-dimensional structures [1,20,21]. PDMS and thermoplastics are commonly used for soft lithography and other mold-based fabrication processes. These methods tend to be more cost-efficient compared to traditional glass or silicon-based fabrication [22,23]. One of the drawbacks of PDMS and PMMA is they tend to have greater surface roughness which can cause issues with layer bonding [19].

**Figure 2:**
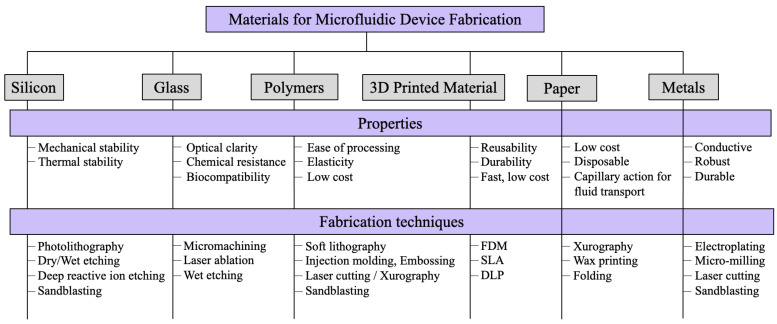
Summary of properties and fabrication techniques based on material choice for microfluidic device fabrication.

Due to the improving resolution and availability of 3D printers, researchers have turned to investigate 3D printed materials for microfluidic channel fabrication. There is a diversity of 3D printing techniques and materials to fit different applications. For fused deposition modeling (FDM) printing, the filament is the required material. Some examples of common filament types include polylactic acid (PLA), acrylonitrile butadiene styrene (ABS), carbon fiber, and thermoplastic polyurethane (TPU). PLA is easy to print and is brittle while ABS is stronger and more heat resistant. Carbon fiber provides greater durability and stability but can be difficult to print, while TPU can create more flexible and impact-resistant parts [24]. For stereolithography (SLA) or digital light process (DLP) printing, resin materials are used. Resins are available with different properties including color, rigidity, heat resistance, and elemental makeup. Three-dimensional printed materials can be used in combination with other materials such as PDMS, acting as a master mold for soft lithography [25,26]. Additionally, the integration of hybrid methods such as combining sandblasting with laser cutting or 3D printing with micromachining further expands the scope of fabricating intricate and multi-material microfluidic devices. Figure 2 categorizes material properties and techniques.

### 2.2. Conventional Fabrication: From Photolithography to Soft Lithography and Beyond

**Figure 3:**
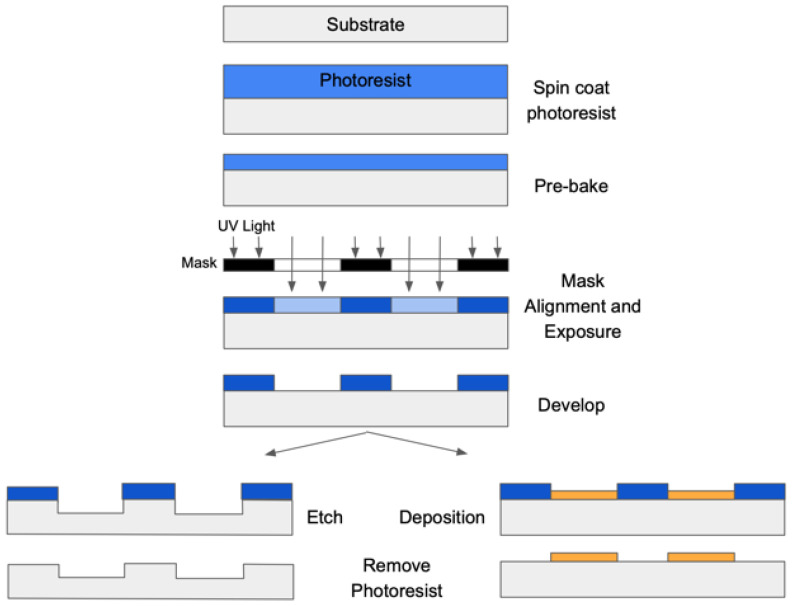
Steps of photolithography followed by etching or deposition of secondary material (yellow).

Photolithography and etching techniques, borrowed from the semiconductor and integrated circuit industries, have long been the cornerstone of microfluidic channel fabrication [2,4,19]. However, these processes come with complexities and requirements due to multi-step processes, required cleanroom environments, and chemical usage [1,18,27]. A detailed description of these steps can be found in Figure 3. The initial step of photolithography involves coating the substrate surface with a photoresist via spin coating. This photoresist is then exposed to ultraviolet light through a mask to create the desired pattern. After exposure, the exposed photoresist is selectively removed, leaving behind the desired pattern. Baking steps are often included both before and after the UV exposure to enhance the process [28]. Subsequently, either a wet or dry etch can be conducted to remove material from the exposed areas, or an additional secondary layer can be deposited to add material to the exposed regions. Following these steps, the remaining photoresist is eliminated, resulting in the final structure. Photolithography offers significant advantages, particularly its ability to achieve high-resolution patterning features. However, due to the small feature sizes involved, this process demands a cleanroom environment and the use of expensive equipment [29].

**Figure 4:**
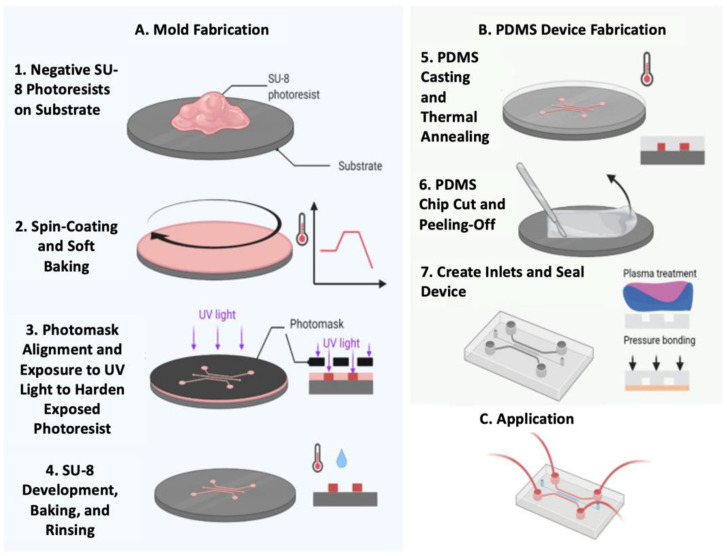
Fabrication of an SU-8 mold and PDMS microfluidic channel using photolithography and soft lithography. Modified from Scott et al. [18]. License: CC BY 4.0.

To address the need for faster fabrication and increased output while reducing cleanroom time, soft lithography techniques have been developed. Soft lithography involves creating a master structure using photolithography, which then serves as a reusable mold for the rapid production of high-resolution structures outside the cleanroom. Figure 4 illustrates the steps involved in creating an SU-8 mold using photolithography, followed by the production of a PDMS microfluidic device from the mold. PDMS is commonly used for molds due to its elasticity, allowing it to conform easily to the surface topography of the master. However, it is important to note that soft lithography still requires a cleanroom and costly equipment whenever a new master is needed.

In addition to soft lithography, other techniques like injection molding and embossing utilize molds to define and create microfluidic channels. While these methods are advantageous for large-volume sample production, they typically involve higher initial costs for mold development and are time-intensive for prototyping purposes.

Some of the main challenges facing microfluidic devices today include standardization in fabrication procedures, scaling up to larger volumes, and cost-effectiveness primarily in the transition from laboratory to commercial products. This is commonly due to the reliance on specific machinery and training, costly materials, and complex processes, making mass production difficult [4,30]. Because of this, researchers have turned to using more accessible materials and tools for rapid fabrication methods of microfluidic channels.

### 2.3. Advancements in Rapid Fabrication Methods

In recent years, rapid fabrication methods such as 3D printing and microcutting have gained significant attention in microfabrication, particularly during the prototyping and research phases of microfluidic channel development. These methods offer fast and cost-efficient solutions for fabricating microfluidic devices without the need for a cleanroom [31].

There are various 3D printing techniques available including FDM, SLA, and DLP. FDM operates using material extrusion and is the most widely used and thought of 3D printing technology. It deposits melted material layer by layer to build up the desired part. FDM parts tend to have rougher finishes and more pertinent layer lines [32,33]. Compared to FDM, resin-based prints are more capable of high accuracy, fine features, and smooth finishes [34,35]. SLA printing uses light-reactive materials that are cured layer by layer into hardened plastic using a laser. This allows SLA to achieve high-resolution parts with a smooth finish [33,36]. Like SLA, DLP 3D printing uses light to cure liquid resins into a hardened part. Rather than using a laser, however, it uses a digital light projector, allowing full layers to be cured at once. DLP printing provides high-quality prints in even less time than SLA. Both SLA and DLP printing require post-processing steps, unlike FDM printing where parts can typically be used right off the build plate [33,37]. Overall, 3D printing techniques allow for the creation of more complex 3D microfluidic channel structures that tend to be more durable and reusable than conventional fabrication methods. Due to this, 3D printed microchannels have been integrated into various areas of research [38,39]. For example, Naghash et al. developed a 3D printed micromixer for improved fluid mixing in lab-on-a-chip applications [40]. Kara et al. demonstrated the use of both SLA and FDM 3D printing for microfluidic chips with 1 mm diameter channels [41]. While 3D printing allows for quick and highly customizable prototyping, its resolution limitations make it unsuitable for smaller microfluidic designs [1,18,42,43]. On the other hand, microcutting methods such as xurography [1,22,44,45] and laser cutting [1,22,46] allow for layer-by-layer or laminate microfluidic fabrication, resulting in consistently fabricated and highly uniform channel thicknesses [18,47,48,49]. These methods are also more accessible and affordable than traditional cleanroom equipment [22,50,51] and can be found at most universities, machine shops, or laboratories [22,52].

Laser cutting stands out among rapid fabrication methods due to its superior cutting resolution. It provides precise and intricate noncontact cutting or etching, allowing for the creation of microfluidic designs with high resolution. It operates by focusing a laser along a path causing the rapid degradation and evaporation of the material. The path is governed by a provided computer-aided design (CAD) and the cutting quality is dependent on the set laser properties: power and speed. Laser cutting equipment comes at a higher upfront cost and requires careful handling and training due to the safety risks associated with lasers [18,53,54]. Xurography, on the other hand, offers a more accessible and cost-effective option for fabricating microfluidic devices. Also referred to as craft/vinyl cutting or razor writing, this method involves using a small knife or blade dragged along the surface to create a desired cutout. It can achieve satisfactory results for many applications, especially those that do not require extremely fine features. Craft cutters are easily integrated into any workspace or lab due to their minimal safety risks, training, and setup [18,55]. Depending on the design complexity, both laser cutting and xurography can create finished products in minutes with high repeatability.

**Figure 5:**
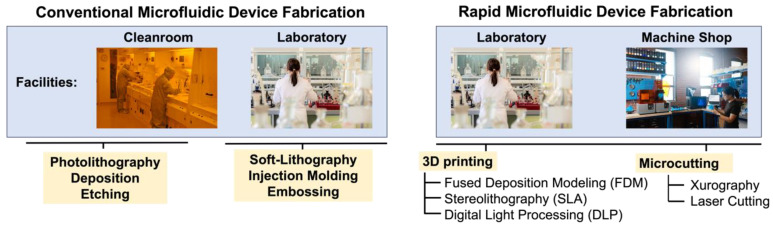
Conventional and rapid microfluidic device fabrication methods. Cleanroom, laboratory, and machine shop images are reproduced from open-access sources with license CC BY 2.0.

One significant advantage of rapid fabrication techniques is the ability to make changes to channel designs quickly and easily. Alterations can be made digitally using CAD software and new structures can be re-fabricated within minutes [49,50,52]. This contrasts with traditional fabrication methods, which often require designing and ordering new masks or molds for each design change. Although rapid fabrication techniques may have lower resolution compared to lithography techniques, their cost and time efficiency make them particularly suitable for the prototyping and research phases. Figure 5 gives an overview of conventional and rapid fabrication methods and Table 1 summarizes microfluidic device fabrication techniques, including the advantages and limitations of each.

### 2.4. Bonding Techniques for Microfluidic Channel Sealing

After fabricating different parts of a microfluidic device, they must be properly bonded together and sealed to ensure leak-free operation. The manipulation of an open channel for bonding without disrupting the integrity of the channels or clogging them can be challenging, but is a fundamental step for the fabrication of microfluidic devices [56,57]. Therefore, various bonding techniques have been studied and used for microfluidic channel sealing. The choice of bonding technique depends on several factors including surface chemistry, material compatibility, optical properties, uniformity, and desired bond strength [56,58].

**Figure 6:**
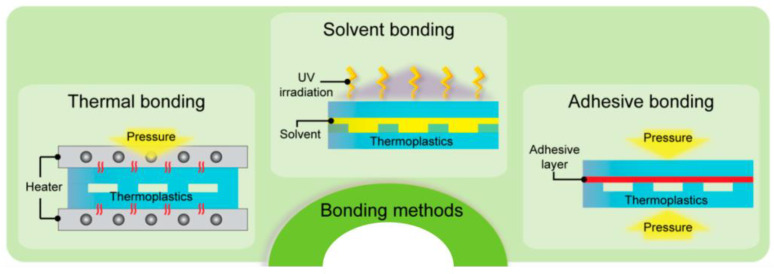
Illustrations of thermal, solvent, and adhesive (double-sided tape) bonding techniques. Reproduced from Trinh et al. [60]. License: CC BY 4.0.

Bonding can be either indirect or direct. Figure 6 shows a summary of these bonding techniques. Indirect bonding uses the addition of another layer or substance to the microfluidic channel surface to establish the bond. Adhesive bonding falls under this category with the use of epoxy, chemical reagents, and adhesive tape. In contrast, direct bonding alters the pre-existing surface to allow bonding to occur without any additional layer. Examples include thermal bonding and solvent bonding [56,59].

Adhesive bonding provides a versatile low-cost solution for joining the two sides of a microfluidic device through indirect bonding. It benefits from its ability to bond almost any structure regardless of surface chemistry or compatibility [56,58]. There are many adhesives available and in general, they can be divided into two categories: liquid and dry. Liquid adhesives include epoxy or glue while dry adhesives can be film or double-sided tape [57,60,61]. Adhesive bonding also benefits from simple curing methods. Most can be cured with time in a room temperature environment, slightly increased temperatures, or in the case of UV glue, with UV light. Some downsides of liquid adhesive bonding include the difficulty in controlling the thickness and uniformity of the adhesive layer. Liquid adhesives applied in excess can clog the channel and both liquid and dry adhesives can trap air bubbles on the surface. The application of adhesives tends to require more manual labor which can be time-consuming and make reproducibility difficult [56,57,59].

Solvent bonding is effective for bonding thermoplastic materials by utilizing a liquid or vapor solvent. The solvent layer creates mobility among the surface polymer chains so that when the two bonding surfaces are brought together, the chains of either side are tightly entangled. When low temperature and pressure are applied, the solvent evaporates, leaving behind a high-strength, physical bond between the surfaces [59,62,63]. Commonly used solvents include various alcohols or acids such as isopropyl alcohol, chloroform, ethanol, acetone, and dichloromethane. A composition of such solvents is commonly used to develop highly effective bonding depending on the materials to be bonded. One crucial consideration is the careful selection of solvent composition to ensure material compatibility and proper evaporation. Excess solvent that remains unevaporated can cause clogging or stress cracks resulting in channel deformation. The distribution of a liquid solvent is also important to provide uniformity in bonding, especially along exposed edges where the solvent can evaporate prematurely [56,59,64]. Vapor solvents help eliminate the issues of clogging and channel deformation since the solvent can evaporate up until the surfaces re-solidify. They also help improve uniformity and surface roughness [59,64].

Another commonly used bonding technique is thermal bonding. Thermal bonding involves heating the surfaces to near or above their glass transition temperatures while pressing them together. These temperatures can range from 50 to 390 °C [59]. This allows the polymer chains between the two surfaces to inter-diffuse and bond together resulting in a permanent seal [56,59]. No intermediate or sacrificial material is required in thermal bonding, making it a simplistic and reliable process. Also, thermal bonding is advantageous for bonding both similar and dissimilar materials so long as they have similar glass transition temperatures. One drawback is that channel deformation and clogging can occur if bonding parameters such as the temperature and pressure are too high. This is due to the increased elasticity and force applied to the channels [58,59,60]. Also, biomolecules immobilized in the channel or on the surface can be affected by elevated temperatures. Proteins, for example, irreversibly denature around 80 °C; if a higher temperature than that is needed for bonding, this will denature the proteins [65].

## 3. Double-Sided Tape: A Versatile and Cost-Effective Approach for Microfluidic Channel Fabrication and Bonding

Over the past two decades, double-sided tape has continued to grow as a common material used for the development of low-cost microfluidic devices due to its ability to define and seal a microfluidic channel. Double-sided tape is attractive to fabricate microfluidic channels because a rapid fabrication method like laser cutting or xurography can be used to define a desired microfluidic channel structure. The tape can be cut to the desired shape and the dimensions of the channel and adhered to a substrate material such as glass or polymer to enclose the channel. Due to the adhesive properties of double-sided tape, it can also be used to reliably seal different layers of microfluidic devices together. Compared to other conventional microfabrication methods and materials, no chemical or thermal treatment is needed. Double-sided tape provides a strong and reliable bond, allowing for easy and quick assembly. It also tends to demonstrate an increase in bond strength over time; therefore, when first placed, it can easily be removed and repositioned to aid proper layer alignment and then will remain strong throughout its lifetime. Proper bonding and sealing are imperative to preventing any fluid leaks or misdirection, ensuring that fluid is flowing only through the desired channels [48,66,67,68]. This can be especially useful when channels or substrates are made from a material that is difficult to bond with other materials.

Another benefit of double-sided tape as an adhesive is how flexible and adaptable it is. It can conform to irregular surfaces allowing for the creation of complex 3D microfluidic devices which could be difficult to fabricate with conventional methods [69]. The use of double-sided tape for creating and bonding microfluidic channels has also led to the use of multiple layers of double-sided tape or other materials being stacked together, adding another degree of freedom to fluid flow. Fan et al. created several microfluidic channel designs using layers of MSB-1001 adhesive, allowing fluid to flow in both the vertical and horizontal directions [70]. This is a common technique used in paper-based microfluidics [71,72,73,74]. Furthermore, microfluidic devices are commonly used for optical detection; therefore, the optical properties of the microfluidic channel are an important consideration in material choice. Like PDMS or glass, most double-sided adhesives are optically transparent [50,75].

Double-sided tape is commercially available with a variety of thicknesses in micrometer scales, material compositions, and adhesive strengths. It is important to evaluate the chemical compatibility of the adhesive, substrates, and chemicals involved. Incompatibility may cause the tape to degrade or lose adhesion leading to leaks or device failure. A vast variety of double-sided tape with varying chemical resistances are available [68,76,77]. Some double-sided tape types are also known to be biocompatible, allowing them to be used for applications with biological materials [50,52,66,78]. Therefore, they have the potential to be a cost and time-effective alternative for fabricating microfluidic channels.

**Figure 7:**
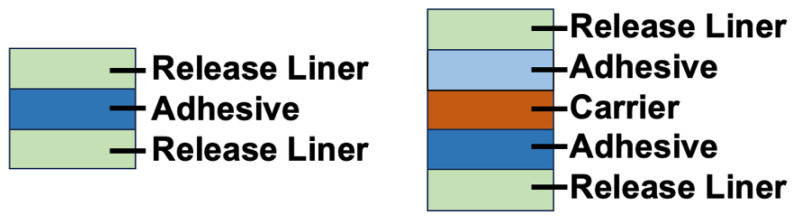
Standard layer stacks for double-sided tape.

Double-side tape commonly consists of either a single pressure sensitive adhesive (PSA) layer sandwiched between two release liners, or two PSA layers separated using a carrier with release liners on the outer ends as shown in Figure 7. The adhesive layer is the layer of sticky material used to join two surfaces while the release liners protect and support the integrity of the adhesive layer prior to use and prevent it from sticking to itself. A carrier layer can be included to separate two different types of adhesive layers, enabling the bonding of two dissimilar surfaces [79,80].

The total tape thickness can typically range from 0.05 to 3 mm based on specific manufacturer and product choice. It is important to choose an optimal thickness based on the desired application because the thickness of the adhesive layer affects the strength and durability of the bond. The height of the microfluidic channel is also determined using the thickness of the double-sided tape. Microfluidic channels made of double-sided tape benefit from being highly uniform which improves the overall adhesion and repeatability amongst samples [48,81]. The adhesive layers tend to be rubber, acrylic, or silicone based. Rubber based adhesives are ideal for indoor environments with little temperature or moisture change. They have a high initial tack meaning they bond very quickly but can easily break down in the presence of solvents or chemicals [82,83]. Due to this, acrylic or silicone-based adhesives are considerably more popular given their high tolerance to temperature and solvents. Both adhesives take longer to achieve their maximum bonding strength but achieve a more permanent and robust bond. In general, acrylic adhesives are initially tackier than silicone and are ideal for temperatures between 0 and 100 °C. Silicone, however, provides a greater long-term bond strength that is resistant to more extreme temperatures both below 0 °C and above 100 °C [82,84,85,86].

### 3.1. Applications of Using Double-Sided Tape

The double-sided tape applications for microfluidic channel fabrication are broken up by tape manufacturer, then tape type, then specific application. Table 2 describes each mentioned tape type and provides material, thickness, and documented fabrication methods.

#### 3.1.1. Advanced Research Double-Sided Tape for Microfluidic Device Fabrication/Bonding

Advanced Research is a commonly used brand of double-sided tape for microfluidic devices that can be cut with xurography or laser cutting. They provide several lines of tape offering different adhesive compositions and thicknesses. Kratz et al. evaluated four biomedical-grade tape types (ARcare 90106, 92712, 90445, (acrylic-based) and ARseal 90880 (silicon-based)), all cut with xurography. It was concluded that ARcare 90106 showcased exceptional structural resolution and ARcare 90445 and 92712 had robust adhesion capabilities against shear and tensile forces. The three ARcare tape types resulted in >85% metabolic activity when placental epithelial cells were placed onto each adhesive, while the ARseal 90880 displayed a lower metabolic activity but had the highest gas and vapor permeability [78].

A comparative study by Dabagi et al. highlighted the biocompatibility of ARcare 90106, ARseal 90880, ARclean 90716, among other double-sided tape from brands like Marian and Scotch. A single microfluidic perfusion channel and lung-on-a-chip assembly (double channel) were both made containing two layers of double-sided tape cut with a cutting plotter. Two layers of adhesive were sandwiched around a porous membrane containing cells with either glass or PDMS acting as the outer layers. The perfusion channel was 1 mm wide and opened to a 5 mm diameter circle acting as a growth chamber. For the lung-on-a-chip microfluidic channel, the width and length were 1 mm and 17 mm, respectively. The ARclean 90716 was able to maintain a fluid-tight seal for 1 week with a continuous flow rate of 10 to 250 µL/min but showed higher cell death for the HBEC-6KT cells. ARcare 90106 and ARseal 90880 showed no impact to cell viability but ARseal 90880 was chosen for that cell type due to it being easier to cut. For another type of cell, Calu-3 cells, ARclean 90716 demonstrated compatibility and high adhesion to PDMS. Overall, this study demonstrated the viability of various types of double-sided tape and developed a technique for future lung-on-a-chip structures and other microfluidic devices [68].

Amongst other tape, ARcare 90106 has been widely used due to its ease of cutting and biocompatibility [68,78,93]. It has successfully been employed in various biosensing applications for bonding substrates such as glass and polymers and for creating microfluidic channels for fluid flow. For example, ARcare 90106 in combination with ARFlow 93049 was used to fabricate an autonomous microfluidic network with xurography for detecting extraterrestrial life markers such as adenosine triphosphate. Soda-lime glass was used as the substrate and was functionalized on one side with a layer of polymer brushes. The ARcare 90106 was placed on top of the polymer brush layer to define the microfluidic network and then sealed with ARFlow 93049. Sample fluids reached chemiluminescent detection sites through capillary flow into eight branches of the microfluidic network. The use of double-sided tape was advantageous for this application due to its room-temperature bonding. This prevented any hindrance to the recognition molecules immobilized at the functional sites prior to the final sealing of the chip [92].

Another group utilized ARcare 90106 to fabricate a 3D microfluidic network for sorting and monitoring the biomass of living cancer cells in a culture medium. Three layers of ARcare 90106 were razor cut and stacked together to create a 3D microfluidic network with a top and bottom channel separated by an intermediate layer, allowing flow between the two channels. The inlet and outlet holes were 5 mm while the separation hole was 3 mm. The channels between these holes were 1 mm wide and 6 mm long. The outer tape layers were bonded to glass substrates containing electrodes. During testing, the ARcare 90106 showed no delamination when pressure was applied, demonstrating its robustness. This device was able to successfully separate micro-beads from deionized water showing the system’s potential for applications like tissue engineering, tumor cell research, and space-based biological monitoring [91].

**Figure 8:**
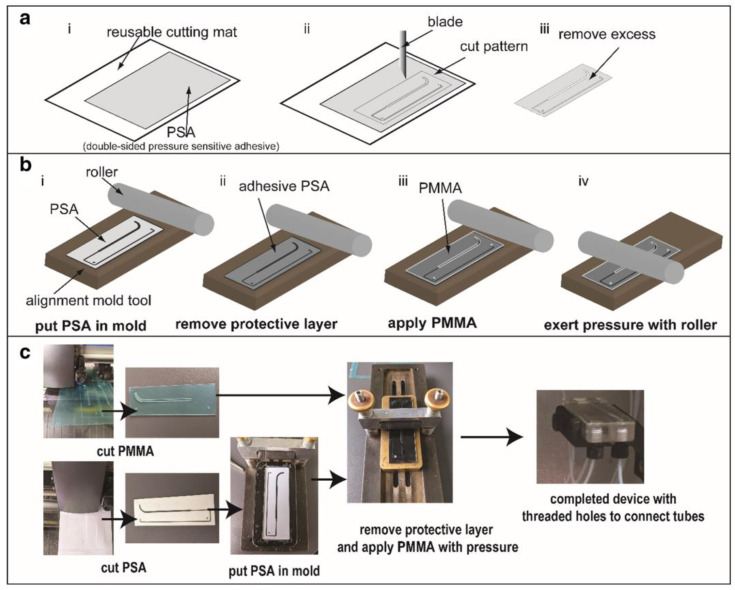
(**a**) Cutting process and (**b**) bonding technique using alignment tool and roller. (**c**) Images of fabrication steps. Open access figure from Wang et al. [95]. License: CC BY 4.0.

Wang et al. fabricated a multilayer 3D microfluidic chip using seven alternating layers of PMMA sheets and ARcare 90106. Both the PMMA and tape were cut with a cutting plotter to define the microfluidic channels. The overall chip structure is shown in Figure 8. The center double-sided tape layer has a 0.7 mm wide channel near the inlet and increases to 1.5 mm in the mixing area. This layer is sandwiched using PMMA layers with similarly cut patterns. The double-sided tape layers for sheath flow are cut and applied to either side of the PMMA layers and then sealed with a PMMA sheet and carrier. A simple lamination process was used for each bonding step by using a roller to exert pressure. This approach was successfully applied to design a 3D hydrodynamic focusing device for the synthesis of varied-size gold nanoparticles, demonstrating the potential of ARcare 90106 for creating complex microfluidic devices efficiently [95].

**Figure 9:**
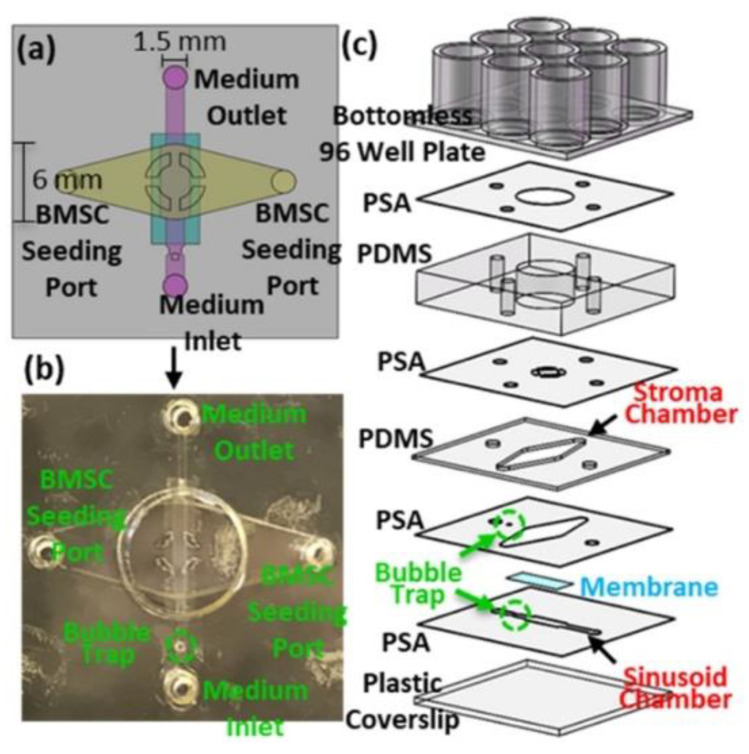
(**a**,**b**) Top view and (**c**) layer stack of microfluidic device with layers of ARcare 90106 and PDMS. Open access figure from Sui et al. [94]. License: CC BY 4.0.

Sui et al. used ARcare 90106 together with PDMS to develop a microfluidic device that simulated key features of the bone marrow environment and provided valuable insights into how cancer cells operate. In this study, both PDMS and ARcare 90106 layers were patterned with a craft cutter with a resolution of 500 µm. This method allowed the entire device to be fabricated in less than two hours. The tape layers bonded each subsequent PDMS layer and acted as fluid pathways between layers as seen in Figure 9. The use of a bottomless 96 well-plate allowed six culture experiments to occur with one device [94].

Ma et al. used laser cut ARcare 90106 to fabricate a microfluidic chip mimicking the shear stress effects on human umbilical vein endothelial cells (HUVEC). A simple three-layer microfluidic chip with a middle layer consisting of laser cut double-sided tape was used for experimentation. The chip contained a single microfluidic channel that was 25 mm long, 0.27 mm wide, and 0.103 mm high. Varying materials were experimented with to act as the top and bottom layers. The top layer was used as a window to view the cell growth and therefore transparent materials like a glass slide, glass coverslip, or polyester slide were tested. Cell growth occurred on the bottom layer; therefore, materials with proper cell adhesion and viability such as glass, permanox, or polystyrene slides were tested. All microchip combinations demonstrated suitable cell attachment and proliferation with permanox and polystyrene substrates having the highest adhesion efficiency of HUVEC. In terms of shear stress, all microchips were capable of withstanding 1 to 4 dyne/cm^2^ [52]. ARcare 90106 was also used by Martinez et al. in a nasal mucosa biosensing device for acute toxicity monitoring. SLA 3D printing was used to construct the chip components and bonded to a PET membrane using the double-sided tape cut with xurography. Overall, this research demonstrated how affordable and rapid fabrication methods can be beneficial as pre-screening tools [96].

Other tape types such as ARcare 8939 and 92712 have been utilized for different microfluidic applications, including a self-powered starch detection device. This device integrated 3D-printed micropumps onto a microfluidic chip. One method for the microfluidic chip fabrication used a cutting plotter to cut the channel into a layer of ARcare 8939. This was adhered to a PMMA substrate and sealed with ARcare 92712. Another mentioned microfluidic chip fabrication used a 3D printed acrylic microfluidic channel with ARcare 8939 as the bottom layer for sealing. For this application, the tape provided a leak-free bond and low-cost fabrication technique between components and microfluidic channel layers. This device was able to successfully demonstrate self-powered microfluidics using rapid fabrication methods for a colorimetric test for starch detection [88].

Similarly, ARcare 8939 and 90880 were also used to investigate the fabrication of self-powered microfluidic devices towards use in a larger fluid volume analysis. ARcare 90880 was used to assemble and seal the micropumps for the microfluidic channels. ARcare 8939 was cut with a cutting plotter to define the microfluidic channel that was adhered to a cyclo olefin polymer (COP) top layer and PMMA bottom layer sealed with another layer of tape. This work demonstrated the benefits of fast prototyping and device design using xurography and materials like double-sided tape. The best micropump from this work reported an autonomous flow of more than 800 µL of sample fluid for 90 min in a single microfluidic channel. It was also used to demonstrate a multi-functional device for blood analysis capable of plasma separation, transport, and storage [89].

The ability of double-sided tape to bond commonly used materials such as PMMA and PDMS was demonstrated in a study using ARseal 90880 and ARcare 8939 where both proved to simplify layer bonding while maintaining proper adhesion. The bonding strategy involved adhering the laser cut double-sided tape to the PMMA layer first and then compressing the layers with a clamp for 2 min. These layers and the PDMS were then exposed to oxygen plasma treatment and adhered all together. Two microfluidic channel patterns were fabricated with this technique. The first was a straight channel with a width of 1 mm and a length of 30 mm. The other was a serpentine pattern with a width of 0.3 mm, a total length of 190 mm, and a gap of 0.4 mm between channels. The average tensile strength for the PMMA-tape bond was around 82 and 73 psi for the ARcare 8939 and ARseal 90880, respectively. For the PDMS-tape bond, the tensile strengths were around 54 and 52 psi. The channels were also able to avoid any leakage or material degradation after being immersed for three weeks with a flow rate of 30 mL/min and obtained a burst pressure of 50 psi [90].

Another capability of laser cutting Advanced Research tape includes the use of ARcare 92712 as a flexible microfluidic channel for a low-cost wearable sweat detector. A 1 mm wide channel with an inlet hole on one side was laser cut into a layer of double-sided tape. This layer was adhered to a polyimide (PI) substrate containing the printed electrode and a polyethylene terephthalate (PET) substrate with an absorbent pad for sweat evaporation. This device allowed for the continuous monitoring of glucose and cortisol on synthetic skin and showed great potential for the future of wearable microfluidic devices used for biomarker measurements [98].

**Figure 10:**
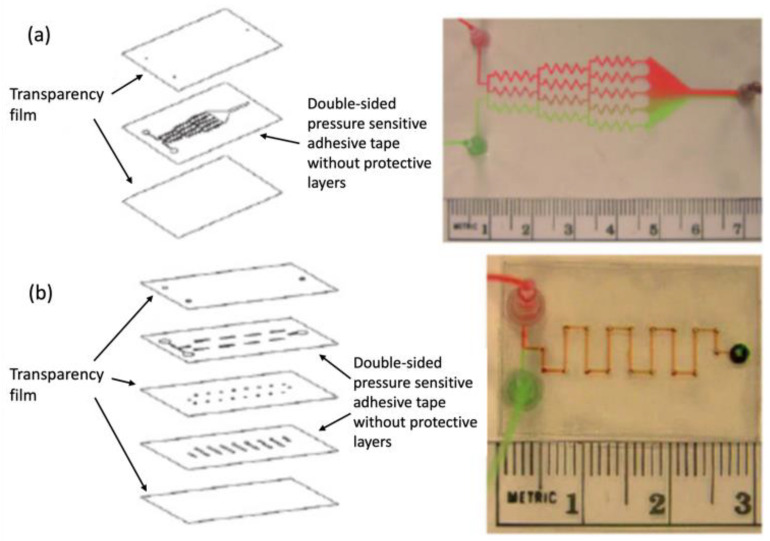
(**a**) Concentration gradient mixer and (**b**) serpentine microchannel mixer fabricated with transparency film and ARcare 8890. Figure courtesy of Yuen et al. [87] with permission from the author.

Goral et al. further demonstrated the potential of ARcare 8890 and xurography-based fabrication by producing a flexible microfluidic device using a desktop digital craft cutter. This allowed the fabrication of microchannels as narrow as 200 µm and facilitated the production of 3D devices using just ARcare 8890 tape and printer transparency film, all within minutes. The fabricated channels were used to create a gradient generator and three-dimensional serpentine microchannel mixer as seen in Figure 10 [87].

ARcare 8890 and 90445 integrated with an optical sensor has been used to observe the adhesion of Salmonella cells on biochip surfaces. Both tape types were laser cut to define the linear microfluidic channels and adhered to a sensor chip. To seal the device, two different top layers drilled with inlet and outlet holes were made of either polyether ether ketone (PEEK) or glass. It was noted that the narrowest channel that could be fabricated with ARcare 8890 was 120 µm and it could be removed from the sensor chip after use with butanone. These devices benefitted from simple fabrication and being fully transparent, allowing successful protein adsorption measurements [50].

**Figure 11:**
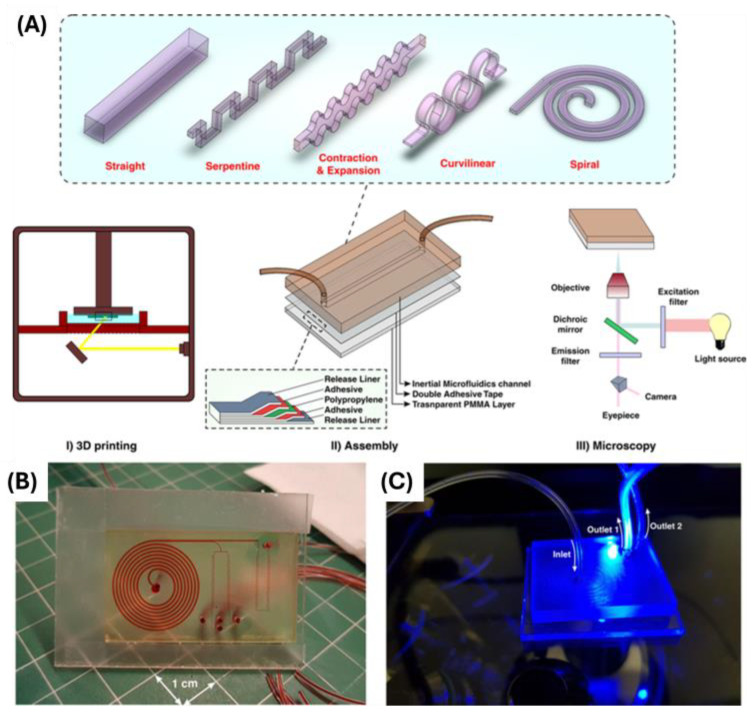
(**A**) The channel designs created using a high-resolution SLA 3D printer and the fabrication steps from 3D printing, assembly with the double-sided tape, and microscopy. (**B**) Finalized product with spiral and serpentine microchannels. (**C**) Fluorescent microscopy. Open access figure reproduced from Bazaz et al. [43]. License: CC BY 4.0.

ARcare 90445 has also been used for adhesive purposes without the need for specific patterning. One study used ARcare 90445 to bond microfluidic channels fabricated with a high-resolution 3D printer to a PMMA sheet. This double-sided tape provided a strong bond capable of sustaining high-pressure fluid flows. Figure 11 shows the various microfluidic channels that were 3D printed and attached using the ARcare 90445 [43].

ARcare 90445 was also used to create an automated droplet microfluidic for producing cell spheroids at higher rates and lower costs without laser cutting or xurography. The holes for the inlets and outlet of the device were cut into the adhesive using a 2 mm biopsy punch. The channels were fabricated in PMMA and then sealed with the ARcare 90445. Due to the high production throughput, low-cost, and automation, this device was successful at generating spheroids and is a potential for drug discovery screenings and future regenerative medicine [97].

ARcare 94199 was used by Shahriari et al. for the low-cost fabrication of an agarose device used for measuring histone levels in plasma. Xurography is used to cut each layer of the device consisting of several layers of adhesives and films. ARcare 94199 in combination with PET demonstrated the best performance when exposed to the dyes and proteins. PVC film, Kapton tape, and single sided ARflow 93049 were also used as layers for constructing the microfluidic device which was shown to be successful at detecting histones [99].

#### 3.1.2. 3M Double-Sided Tape for Microfluidic Device Fabrication/Bonding

3M is another prominent manufacturer of double-sided tape extensively used in microfluidic device fabrication. 3M 55257 was used by Ragavendar et al. to develop low-cost microfluidic chips for point of care (POC) nucleic acid testing. A digital craft cutter was used to cut the microfluidic chip pattern into the tape such that 35–40 mL of sample fluid could be accommodated. It was then sealed between two transparent sheets, aligning and punching holes at the fluidic channel’s entry and exit points. The microfluidic devices were evaluated with a VERSANT sample preparation reagent kit to observe and compare DNA yield from blood samples. The microfluidic device was able to achieve an acceptable DNA yield and quality [115].

Another tape type from 3M, 300LSE, was used to create microfluidic channels for electrochemical devices on transparent polyvinyl chloride (PVC). A linear microfluidic channel 39 mm in length and 450 µm wide was cut into the 300LSE with inlet and outlet holes of diameter 3.14 mm using a knife cutter. Graphite pencils were inserted into the knife cutter and used to draw the electrodes onto the bottom PVC substrate. The 300LSE microfluidic channel was adhered to the bottom PVC layer containing the electrodes and sealed on top with another layer of PVC containing inlet and outlet holes. As proof of concept, this device was used to detect ascorbic acid in common drugs and was therefore shown to be a successful low-cost device for electrochemical analysis [101].

Lin et al. reported fabricating complex 3D structures for wearable microfluidics using 300LSE for the observation of molecules from sweat. By combining multiple layers of laser cut tape and transparent PET film sheets, the flow could be controlled in the horizontal and vertical direction for spatially efficient 3D microfluidic devices. Three microfluidic channel designs and devices were fabricated and used for successful sweat collection, filtration, and simultaneous actuation and sensing [103].

300LSE was also used as a part of an educational microfluidic assembly kit for seventh and eighth-grade students to teach the basics of microfluidic devices and experimentation. The kit cost under USD 2 of materials and provided students the ability to fabricate a droplet mixer, introduce the microorganism *Euglena gracilis* into the system, and observe their phototaxis. Each kit used acrylic and laser cut 300LSE as the microfluidic building blocks along with the necessary inlet and outlets, syringes, and provided fluids and organisms [102].

**Figure 12:**
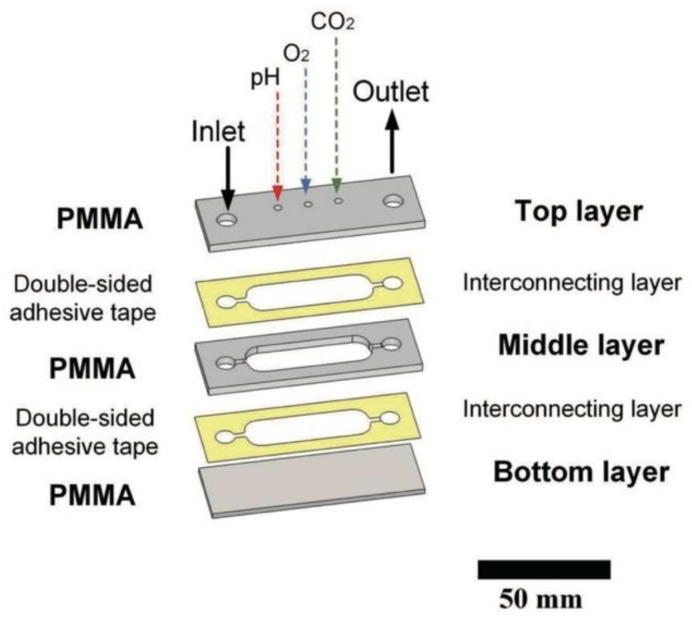
PMMA and 3M 9088 tape layered structure for saliva measurements. Figure courtesy of Podunavac et al. [111] with permission from the author.

Podunavac et al. used 3M 9088 cut with a plotting cutter to bond PMMA layers to develop a microfluidic device for measuring saliva parameters. As seen in Figure 12, PMMA made up the top, middle, and bottom layers. The top and middle PMMA layers were cut with a CO_2_ laser to create the microfluidic channel, inlet/outlet, and sensor attachment holes. Two interconnecting layers made up of the tape were cut with xurography in the channel pattern to bond either side of the middle PMMA layer. The tape was shown to be a reliable adhesive with no leakage after cold lamination with the PMMA layers. This device demonstrated the ability to combine rapid fabrication methods with biocompatible and transparent materials like PMMA and 3M 9088 to create precise and readily integrated microfluidic systems. Using commercial PreSens sensors, this device was used to measure pH, O_2_, and CO_2_ from saliva samples [111].

Hwang et al. experimented with three different types of double-sided tape for the microfluidic channel construction: 3M 309, 3M 9495MP, and T-#7720 from Tapeworld. These were to be used in a polymerase chain reaction (PCR) chip. The proposed structure included four layers. The bottom was a PCB containing the heating circuit and thermal sensor. To prevent molecules from sticking to the PCB, a layer of 3M 309 was used as an intermediate layer. Above that, the double-sided tape layer containing the microfluidic channel was placed. The channel design was constructed using a cutting plotter. To seal the channel and create the inlet and outlet, a polypropylene film was used as the top layer. Each tape was tested to observe its ability to withstand the high temperatures (95 °C) and pressures associated with the PCR process. They also needed to have little to no adsorption of the fluorescent reagent. All were able to withstand the required temperature and pressure and showed similar adsorption. The height of the chip was required to be 400 µm, therefore the thickest tape, T-#7720 (200 µm), was chosen to minimize the required number of layers. After testing, this micro-PCR chip showed A comparable performance to conventional PCR processes while reducing overall chip cost [104].

3M Very High Bond (VHB) F9460PC double-sided tape was used by Lucas et al. in a low-cost passively pumped microfluidic device for cellular analysis. Computational fluid dynamics simulations were performed to determine the optimal channel shape and width for efficient fluid mixing and velocity. The channel was therefore designed to be 10 mm long and 4.5 mm wide and cut out of the double-sided tape using xurography. 3M VHB F9460PC was chosen over other tape types because it demonstrated increased imaging signal, stability, and lack of leaching. This work also commends the uniform thickness, inertness, and non-autofluorescence of the tape. Furthermore, it was important that the tape could maintain good adhesion and performance in wet conditions. The cut double-sided tape was adhered to a glass slide base. To seal the top, a glass coverslip covered the center of the channel while two PDMS pieces covered either end of the microfluidic channel containing inlet and outlet ports. With this passively pumped microfluidic device cyclic imaging of immune and tumor cells were performed at significantly lower experimentation and fabrication costs [81].

**Figure 13:**
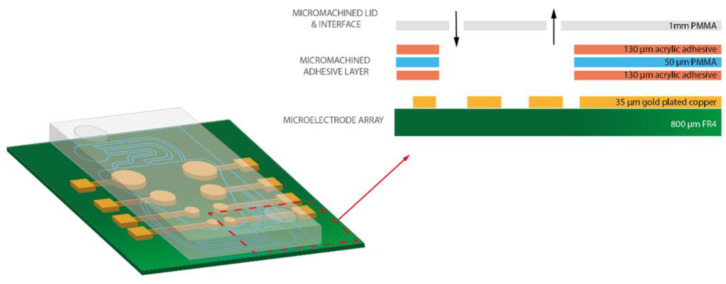
Three-dimensional model of microfluidic channel layers. Open access figure courtesy of Ribeiro et al. [110]. License: CC BY 4.0.

The most used 3M adhesive for microfluidic fabrication is 468MP. It can easily be purchased in large sheets making it readily accessible and cuttable. This tape has been used in several organ and lab-on-a-chip devices. As shown in Figure 13, Ribeiro et al. fabricated a brain-on-chip device using 468MP and PMMA. Both the PMMA and the double-sided tape were laser cut and adhered to a multi-electrode array. The device was able to achieve a flow rate of 80 µL/min with minimal background noise and is a promising step towards improved brain-on-chip devices with continuous flow [110].

Similarly, 468MP has been used to develop a smartphone-based surface plasmon resonance system for biodetection. This device consisted of laser cut acrylic and 468MP that were adhered together and integrated onto a Blu-ray disc chip. This device was able to demonstrate the real-time monitoring of bovine serum albumin (BSA) and the biodetection of rabbit anti-mouse IgG antibody with a dynamic range from a few nanomolar to micromolar concentrations [109].

Biosensing capabilities with 468MP was also demonstrated for a wearable glucose monitor for minimally invasive diabetes monitoring. The device consisted of a sensing, fluidic, and adhesive layer. The adhesive layer consisted of the laser cut 468MP and PMMA film and was sandwiched between the sensing (electrode) and fluidic layers. The fluidic layer was fabricated using photolithography on a thin flame retardant four layer and the adhesive layer was laser cut to match the microfluidic channel pattern. This device was able to successfully perform glucose detection reaching a limit of detection of 2 µM for a flow rate of 10 µL/min [107].

Panraksa et al. used 468MP to detect lipoarabinomannan (LAM), a urinary biomarker indicative of tuberculosis. 468MP was chosen due to its high chemical resistance and laser cut to define the microfluidic channels. Polyester film was adhered to the double-sided tape on either side. When tested, the limit of detection for LAM was found to be 31 ng/mL. Urine samples spiked with LAM were also tested for device validation and proved successful [76].

468MP has also been used to monitor the response of genetically modified helicobacter pylori bacteria to exogenous factors. The tape was laser cut into two channels allowing the simultaneous monitoring of the control and test samples and adhered to PMMA and a glass slide. Fluorescence changes in the channel were used to monitor the bacteria’s response to stimuli such as acidity. Overall, this device was shown to be a simple yet reliable method for long-term experiments of gene expression and cell metabolism [105].

Portable electro-wetting on dielectric (EWOD) microfluidic devices have also been improved with the use of laser cut 468MP. Maintaining the distance between the ground plane and electrode pads is an important factor in EWODs, therefore a double-sided tape was an advantageous solution due to its fixed height. Two layers of 468MP were laser cut to contain the media around the electrode pad and maintain the proper height. This EWOD system is capable of DNA isolation protocol and could be easily integrated into a hand-held POC device for future applications [108].

**Figure 14:**
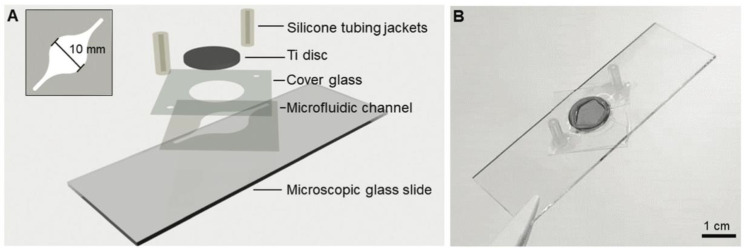
(**A**) Layer stack showing the use of 3M 468MP as the microfluidic channel and (**B**) photograph of the finished product. Figure from Carter et al. [106]. License: CC BY-NC 3.0.

Microfluidic channels made of laser cut 3M 468MP can also be fabricated. 3M 468MP and PDMS were compared by Carter et al. for evaluating the biological properties of biomaterials, specifically a titanium alloy for bone repair. The PDMS channel was fabricated with soft lithography and connected to the titanium disc with an additively manufactured fixture, as seen in Figure 14. The 3M 468MP channel was cut using a cutting plotter with a length of 26 mm and a width of 10 mm. It was sandwiched between a glass slide and cover glass with the titanium disc affixed above the cover glass with an optical adhesive. The inlet and outlets were connected to silicone tubing jackets. When comparing material inertness and availability as well as fabrication ease and reliability, the double-sided tape-based device was preferred. MC3T3-E1 cells were successfully grown on the double-sided tape-based device and tested for cell viability, proliferation, and differentiation over 10 days [106].

Other types of 3M double-sided tape have been employed as well. 467MP and laminated transparency film were used by Jang et al. for a pump-free paper-based microfluidic device for rapid mixing. The device consisted of five layers with alternating 467MP and transparency film. The double-sided tape and one layer of transparency film were laser cut to define the channels of 3 mm wide each. The design consisted of two inlets with channels that merged and reached a fan-shaped paper. The device was able to successfully detect organophosphate pesticide on food by mixing the pesticides with the enzyme and indicator to provide colorimetric readings of pesticide levels. A smartphone application was also developed by Jang et al. allowing one to take a picture of the fan-shaped paper and receive the pesticide concentration based on the paper fan color composition [71].

Another paper-based microfluidic device using 3M 9965 developed a fluorescence immunoassay tested with C-reactive protein (CRP). The device structure from bottom to top consisted of cartonnage paper, water-resistant paper, 3M 9965, and cellophane film. The microfluidic channel laser cut into the double-sided tape had a width of 1 mm and a length of 11 mm. The top cellophane film was also patterned with 1 mm diameter inlet and outlet holes. CRP was able to be detected with this device with a limit of detection of 10–20 ng/mL which is satisfactory for high-sensitivity CRP detection. This device also benefitted from being made with 93% cellulosic material, making it a sustainable device that can be disposed of safely with little environmental waste [113].

3M 9965 has also been used to integrate with electrochemical sensors. This device consisted of electrodes patterned on a glass substrate with a microfluidic pattern defined by the laser cut double-sided tape and sealed with a cyclic olefin copolymer (COC) slide containing the inlet and outlet holes. A peel test quantified the adhesion strength of 3M 9965 to be 0.18 and 0.21 N/mm to the glass and COC substrate, respectively. The strong adhesion of the double-sided tape ensured no leakage, delamination, or channel rupture for commonly applied microfluidic pressures, with a flow rate of up to 5000 µL/min [48].

Similarly, 3M 9965 has been used to create reconfigurable microfluidic building blocks for the high-throughput screening of nonhormonal contraceptives. The system consisted of three building blocks. The top layer was the microfluidic channels for liquid handling. The middle layer was a multi-well array with membrane filters while the bottom layer was for waste collection and oocyte culture. The double-sided tape was laser cut and used to adhere layers of PDMS, acrylic, and silicone rubber. This device showed the potential for various microfluidic functions (gradient generators, emulsion generator, and passive values) due to its interchangeable building blocks. A cumulus oocyte complex (COC) expansion array was able to be successfully performed to demonstrate its applicability for nonhormonal contraceptive screening [112].

**Figure 15:**
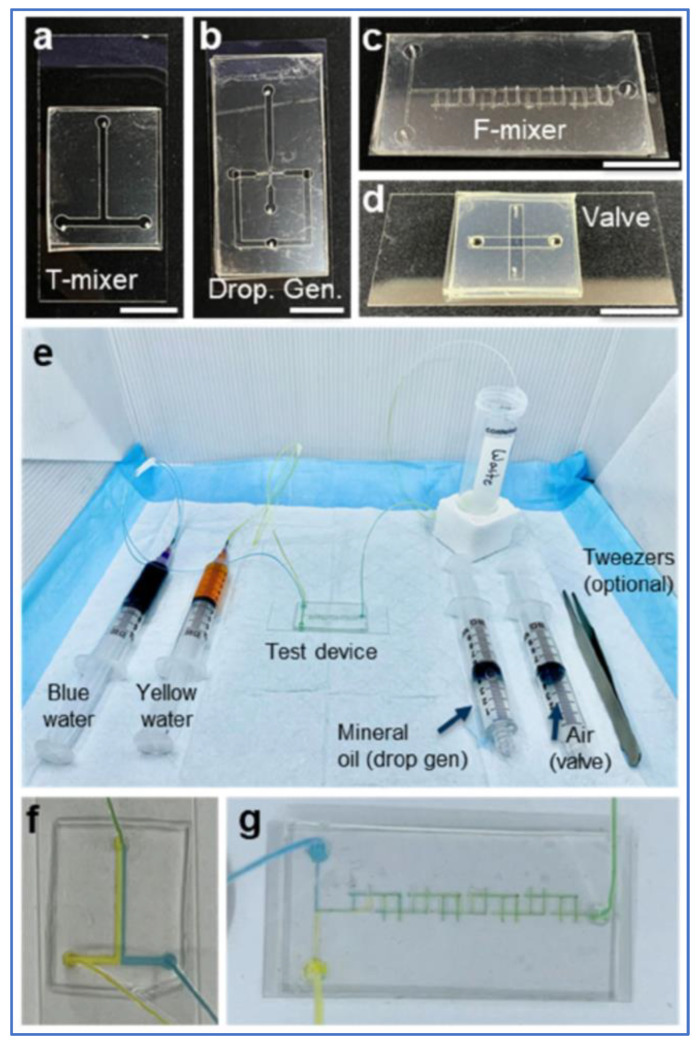
(**a**–**d**) Four microfluidic devices that could be constructed for student activity; (**e**) included fluids and equipment for teaching microfluidic basics; (**f**,**g**) visualization of T-mixer and F-mixer in operation. Figure courtesy of Delgado et al. [114]. License: CC BY-NC-ND 4.0.

Likewise, another low-cost microfluidic assembly activity was created to educate college undergraduate and graduate students on the fabrication and principles of microfluidic devices as seen in Figure 15. This activity allowed for the assembly of four different microfluidic devices: T-mixer, droplet generator, F-mixer, and valve. Each device comprised acrylic, laser cut 3M 96042 tape, and PDMS, allowing students to quickly assemble and test basic microfluidic designs without costly materials, training, or equipment [114].

#### 3.1.3. Other Double-Sided Tape Brands for Microfluidic Device Fabrication/Bonding

Aside from Advanced Research and 3M, other brands of double-sided tape have demonstrated successful use in microfluidic device fabrication with xurography. For example, Montex DX1 has been used to fabricate microfluidic channels to study reaction kinetics and measure dissolution rates of a calcite window. A microfluidic channel with a length of 10 mm and width of 250 µm was cut into the double-sided tape with a cutting plotter. Holes with a 2 mm diameter were also cut on each end of the channel. This tape was adhered to the calcite window and plexiglass to create an air-tight seal that showed no signs of leakage before or during testing. The tape was also able to be re-heated causing its bonds to be weakened so the device could be taken apart as needed. PDMS and parafilm were also tested as potential materials for the microfluidic channel. The PDMS channel was obtained through soft lithography but had difficulty bonding to the calcite. The parafilm could be cut with xurography but had to be heated resulting in channel deformation. Therefore, the double-sided tape proved to be the most reliable and efficient choice for fabrication of the microfluidic device. This device was used to demonstrate in situ dissolution measurements for calcite and was shown to be comparable to that of conventional testing [44].

Another brand, Nitto D5331, was used for defining the microchannels of a nanoplasmonic sensor used to detect the GTF2b protein, relating to colorectal cancer. The tape was cut with a cutting plotter and adhered between a treated Blu-ray disc base and PDMS cover layer with inlet and outlet connections. The channel had a 10 mm length, 1 mm width, and 50 µm height. This device was able to perform real-time detection of the GTF2b protein with a limit of detection of 22.6 pM while benefiting from a highly reproducible and simple fabrication process. This device is promising for future developments in POC plasmonic biosensing [116]. Another type, Nitto 5302A, was found to be most effective for bonding to PDMS upon applying a flame treatment for future organ-on-chip devices. Singer et al. compared four types of tape: Nitto 5302A, ARclean 90176, ARcare 92712, and Scotch 7951. PDMS molds were fabricated with photolithography and each double-sided tape was cut using a plotting cutter. A butane torch was used to heat up both the PDMS and adhesive surfaces for 5 and 3 s, respectively, at around 1300 °C. The Nitto 5302A resulted in the highest burst pressure (325 kPa) and sustained a 3-week Calu-3 cell culture [117].

The common household tape brand, Scotch (developed by 3M), was used to construct a self-pumping mixer for microfluidic devices using various herringbone grooves laser cut into glass. The Scotch tape was used to both define the microfluidic channel and adhere the top and bottom glass substrates together. The channel was laser cut to form a 3 mm wide Y-shaped channel over the herringbone structures. This device structure was able to successfully enhance fluid mixing for capillary flow and was demonstrated with the detection of Ni(II) for environmental applications and enzymatic reaction of hydrogen peroxide and horseradish peroxidase [119].

**Figure 16:**
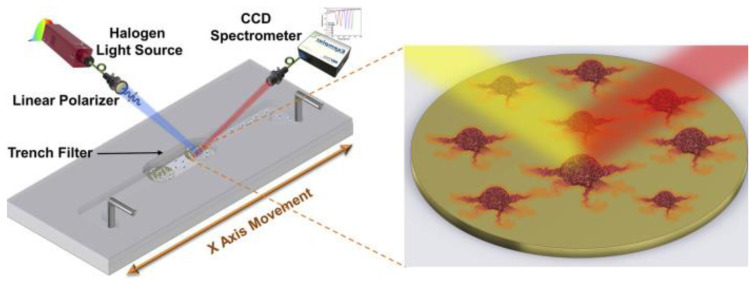
Schematic representing the final microfluidic channel and cell monitoring system. Figure courtesy of Vila et al. [118]. License: CC BY 4.0.

Another brand of medical grade double-sided tape from Microfluidic ChipShop in Germany, Mcs-foil-008, was used to begin the development of a plasmonic biosensor for monitoring cell adhesion and growth using a double-sided tape microfluidic channel as seen in Figure 16. The double-sided tape was laser cut and consisted of a single channel. It was also used to bond the PMMA top layer to the plasmonic biosensor. This study specifically focused on human retinal pigmented cells and was used to monitor cell adhesion. It shows great promise for easily integrated biosensors for future cell monitoring and sensing [118].

Bio-Rad Laboratories have a line of double-sided tape for sealing PCR well plates that can also be applied to microfluidic devices. One study by Fan et al. used MSB-1001 adhesive from Bio-Rad Laboratories to design various multilayer microfluidic devices demonstrating the ease of fabrication and application. MSB-1001 consists of an adhesive layer surrounded by a paper-based protective layer and base polyester layer. This tape was chosen due to its biocompatibility, optically clear nature, temperature resistance, strong adhesion, and low-cost. Various types of microfluidic devices were able to be fabricated with multiple layers of laser cut MSB-1001 adhesive including different droplet generators and diffusion mixers with multiple channels. [70].

One of the first paper-based microfluidic devices was developed in 2007 by George Whitesides and his Harvard research group. For this device, stacked layers of laser cut patterned paper and ACE plastic carpet tape 50106 were used to create 3D microfluidic channels capable of testing various analytes without expensive materials or fabrication processes. The cost of materials for one device was estimated to be around USD 0.03. The channel design was 800 µm wide and 5 cm long, with eight connections between the top and bottom layers allowing fluid flow in both the vertical and horizontal directions [73].

Following this, another paper-based microfluidic device was developed with the same tape type that allowed a user to press a physical ‘on’ button to connect adjourning channels, causing fluid movement across the device. Layers of chromatography paper were patterned with photolithography and alternated with laser cut double-sided tape. The stack was then compressed after layering with a manual press, with a pressure around 20 kg/cm^2^. This device was then able to be used for urinalysis, allowing the user to choose a combination of assays to test for glucose, protein, ketone, or nitrite presence in artificial urine samples [74].

Not every microfluidic device requires sophisticated shapes cut using a craft or laser cutter. Some applications have shown the use of double-sided tape in microfluidic devices without fabricating specific channel designs and simply cutting them by hand or using a hole puncher. For example, various Scotch tape types (Magic, Permanent Double-Sided, MultiTask) were investigated as a potential solution for PDMS bonding and all were shown to be successful at providing a strong bond after being applied and heated, but the Permanent Double-Sided tape proved to be the strongest. The tape acted as the bottom surface of the channel and therefore was used in the shape of the rectangular substrate base. This device consisted of just two layers, PDMS and tape, and was baked for 2 h after adhesion to increase bond strength. The biocompatibility of the various tape types was tested using droplet PCR [66].

Similarly, an adhesive from Excel Scientific, ThermalSeal RTS, was used to investigate its bonding strength with various substrates. This adhesive was cut into a rectangular shape to individually seal and adhere to the microfluidic channels that were patterned in either PDMS, COC, PMMA, PC, glass, and a 3D printed substrate. Each bonded channel was tested for the maximum burst pressure and were all shown to resist pressures over 5 bar except the PDMS-tape and glass-tape bonds which reached 4 and 2.6 bar, respectively. The biocompatibility of the tape was tested with cell cultures. Cell growth up to 72 h after showed equivalent adhesion and growth rates on the tape as compared to on the glass. Fluorescence imaging was also able to be successfully performed with this simple microfluidic device structure [67].

The ability of double-sided tape to bond with PMMA to create reliable microfluidic channels was demonstrated using an optical adhesive from Applied Biosystems, type 4360954. This tape was cut in a simple rectangle to enclose the Y-shaped microchannel patterned in PMMA. For bonding, the PMMA was heated first then adhered to the tape with a pressure of 2 bar for 2 min. The total cost of the device was around USD 1 and required around 90 min for fabrication. This device was then used for the fluorescence detection of antibiotic resistance gene sequences and sequence-specific DNA capture and labeling [120].

### 3.2. Discussion

While there are many advantages of using double-sided tape for microfluidics, there are some limitations based on tape type that should be considered. Microfluidic devices used or fabricated in extreme temperature or pressure environments may exceed the limitations of double-sided tape. In extremely cold temperatures (around 0 °F), the tape can harden and lose its wettability or stickiness. In high temperatures, double-sided tape softens and increases flexibility, which is good for adhesion but detrimental to maintaining the channel structure. In general, most double-sided tape performs best between 59 and 95 °F, but there are tape types designed for high or cold temperatures based on desired applications. For example, the 3M 100MP high-performance adhesives can operate at 300 °F and have short-term tolerance up to 500 °F. The 3M VHB tape series is another option for applications requiring extreme temperatures [121,122]. The effectiveness of double-sided tape is also dependent on the pressure applied upon adhesion. Insufficient pressure can result in areas of little to no adhesion which could lead to leaking in the case of microfluidics. After initial contact, double sided tape tends to increase its bond over time, so allowing sufficient time between initial adhesion and use can mitigate adhesive failures [123].

Another limitation is created by the laser or craft cutter. Depending on the desired channel dimensions, laser or craft cutters are limited by the diameter of the laser and the quality of the cutting knife and may not be able to reliably produce small scale features. The feature size and accuracy of both laser and craft cutters depend highly on the device manufacturer and laser or blade properties. For laser cutting, the spot size of the laser determines the minimum feature size since cuts cannot be made that are smaller than the diameter of the laser. The spot size is based on the focal length of the laser’s focusing lens so smaller spot sizes have more energy per area. Most commercial laser cutters use CO_2_ lasers which typically have minimum spot sizes around 75–120 µm [124]. For xurography, the blade used to cut the material determines the cutting resolution. Cutting blades are available in different angles (30°, 45°, and 60°), widths, and shapes depending on the cutting material. Thin materials like double-sided tape are best cut with a 45° blade [55]. It is also important to maintain a sharp blade to ensure clean cuts. Cutting settings such as speed, power, and force can also be optimized for both laser cutting and xurography to help produce the highest quality cuts. If the speed or power is too great then the cuts may not be as clean or accurate due to material tearing or missed features [22,51].

## 4. Conclusions

As interest and research in microfluidic devices continue to rise, the ability for devices to be low-cost and easy to fabricate has created an incentive for the transition towards more rapid fabrication methods and cost-efficient materials. Double-sided tape is the ideal alternative. It is easily accessible with a variety of thicknesses, material compositions, and adhesive strengths from brands like Advanced Research, 3M, and Scotch. Double-sided tape is optically transparent and inert while providing a uniform thickness. Many are also biocompatible for uses in biosensing, cell cultures, and lab or organ-on-a-chip applications. Some of the more commonly used and characterized biocompatible double-sided tape types are ARcare 90106, 90445, 92712, and 3M 468MP. Another important benefit of double-sided tape is the ability to strongly adhere to any surface. Unlike other bonding techniques, double-sided tape is compatible with all surfaces allowing leak-proof bonding between similar or dissimilar microfluidic channel halves.

Double-sided tape can be patterned to define microfluidic channels using rapid fabrication methods such as laser cutting or xurography. This expedites channel prototyping and production by replacing the complex processes and cleanroom environment required by conventional microfabrication techniques. Laser cutting tends to have greater resolution capabilities than xurography. However, it requires more costly equipment and intensive safety training. Xurography techniques with a craft or vinyl cutter can easily be implemented in any space with low risk. Both rapid fabrication techniques provide a high throughput of microfluidic channel designs that can be easily created with any CAD software for high accuracy and repeatability.

Double-sided tape can provide the architecture for both simple and more complex microfluidic devices by defining and bonding microfluidic channels. There is an endless combination of rapid fabrication methods and double-sided tape choices allowing them to be applied in fields such as medical, biological, chemical, pharmaceutical, and beyond. The integration of double-sided tape advocates for more time spent on microfluidic channel or microfluidic device use and less on fabrication; the same goes for the amount of money spent. Enabling fast fabrication and prototyping abilities can expedite the work in microfluidic devices being carried out so more research papers can be published and more knowledge can be discovered. Beyond that, it will allow more microfluidic devices to successfully transition from laboratories to commercial products in the future. Low-cost, simple to implement materials like double-sided tape can help provide the jump to commercialization by fast tracking prototyping stages and simplifying fabrication to allow high volume production through automation and assembly lines. Double-sided tape is one way researchers can continue to advance the expansion and future of microfluidics, allowing more life-saving devices, instrumentation, and knowledge to be in the hands of patients and medical professionals alike.

## Figures and Tables

**Table 1:** Advantages and limitations of various chemical and mechanical microfluidic device fabrication methods.

	Advantages	Limitations
Chemical		
Photolithography and Etching	High resolution (nm) and repeatability	New mask per design, requires cleanroom, multi-step process
Soft lithography	High-resolution, fast fabrication replicas	Only master copy requires cleanroom, pattern deformation over time, lower aspect ratios
Mechanical		
Micromachining and Microcutting	Fast (sec), design flexibility, no cleanroom required	Lower resolution (mm), tools can wear over time, less clean surface finish
3D Printing	Fast (min), design flexibility, capable of more complex channel profiles and paths	Lower resolution (mm). poor minimum feature size (mm), restricted to resin and filament materials
Embossing	High throughput, design flexibility, high precision, less stress on the material than injection molding	Restricted to thermoplastic materials, expensive and costly molds
Injection Molding	High throughput and design flexibility, high precision	Restricted to thermoplastic materials, expensive and costly molds

**Table 2:** Summary of each referenced tape type and its properties including adhesive thickness and material, brand, and cutting method.

Referenced Tape Types and Properties							
				Fabrication Method			
Brand	Type	Material	Thickness (µm)	Xurography	Laser Cutting	Other	Ref.
Advanced Research	ARcare 8890	acrylic	12.7	✓	✓		[50,87]
	ARcare 8939	acrylic	25	✓	✓		[88,89,90]
	ARcare 90106	acrylic	58	✓	✓		[52,68,78,91,92,93,94,95,96]
	ARcare 90445	acrylic	28	✓	✓	✓	[43,50,78,97]
	ARcare 92712	acrylic	18	✓	✓		[78,88,89,98]
	ARcare 94119	silicone	46	✓			[99]
	ARclear 8932EE	silicone	51		✓		[100]
	ARseal 90880	silicone	46	✓	✓		[68,78,89,90]
3M	VHB F9460PC	acrylic	50	✓			[81]
	300LSE	acrylic	200	✓	✓		[101,102,103]
	309	acrylic	50	✓			[104]
	467MP	acrylic	58		✓		[71]
	468MP	acrylic	132	✓	✓		[48,76,105,106,107,108,109,110]
	9088	acrylic	94	✓			[111]
	9495MP	acrylic	74	✓			[104]
	9965	acrylic	18		✓		[48,112,113]
	96042	silicone	102		✓		[114]
	55257	polyester	12	✓			[115]
Montex	DX1	polyacrylate	70	✓			[44]
Nitto	D5331	acrylic	70	✓			[116]
	5302A	acrylic	60	✓			[117]
Tapeworld	T-#7720	acrylic	200	✓			[104]
Microfluidic Chip Shop	MCS-foil-008	acrylic	140		✓		[118]
Bio-Rad Laboratories	MSB1001	polyester	200		✓		[70]
ACE	Carpet Tape 50106	plastic	60		✓		[73,74]
Scotch	Magic	acrylic	63.5			✓	[66]
	Double-Sided	acrylic	76.2			✓	[66,119]
	MultiTask	acrylic	58.4			✓	[66]
Excel Scientific	ThermalSeal RTS	silicone	100			✓	[67]
Applied Biosystems	4360954	polyester	150			✓	[120]

## Data Availability

No new data was created or analyzed in this study. Data sharing is not applicable to this article.

## References

[1] Niculescu A.G., Chircov C., Birca A.C., Grumezescu A.M. (2021). Fabrication and Applications of Microfluidic Devices: A Review. Int. J. Mol. Sci..

[2] Fiorini G.S., Chiu D.T. (2005). Disposable microfluidic devices: Fabrication, function, and application. BioTechniques.

[3] Whitesides G.M. (2006). The origins and the future of microfluidics. Nature.

[4] Convery N., Gadegaard N. (2019). 30 years of microfluidics. Micro Nano Eng..

[5] What Is the History of Microfluidics?. https://www.fluigent.com/resources-support/expertise/expertise-reviews/what-is-microfluidics/what-is-the-history-of-microfluidics/.

[6] Kumar A., Parihar A., Panda U., Parihar D.S. (2022). Microfluidics-Based Point-of-Care Testing (POCT) Devices in Dealing with Waves of COVID-19 Pandemic: The Emerging Solution. ACS Appl. Bio Mater..

[7] Jamiruddin M.R., Meghla B.A., Islam D.Z., Tisha T.A., Khandker S.S., Khondoker M.U., Haq M.A., Adnan N., Haque M. (2022). Microfluidics Technology in SARS-CoV-2 Diagnosis and Beyond: A Systematic Review. Life.

[8] Berkenbrock J.A., Grecco-Machado R., Achenbach S. (2020). Microfluidic devices for the detection of viruses: Aspects of emergency fabrication during the COVID-19 pandemic and other outbreaks. Proc. Math. Phys. Eng. Sci..

[9] Global Microfluidics Market Size, Share & Growth Report. 2030. https://www.marketsandmarkets.com/Market-Reports/microfluidics-market-1305.html.

[10] Microfluidics Market by Product, Application, End User & Region—Global Forecast to 2026. https://www.marketsandmarkets.com/Market-Reports/microfluidics-market-1305.html.

[11] Sia S.K., Kricka L.J. (2008). Microfluidics and point-of-care testing. Lab Chip.

[12] Future of Medicine: Lab-on-a-Chip Devices Starting to Make an Impact. https://www.nhlbi.nih.gov/news/2021/future-medicine-lab-chip-devices-starting-make-impact.

[13] Yang S.M., Lv S., Zhang W., Cui Y. (2022). Microfluidic Point-of-Care (POC) Devices in Early Diagnosis: A Review of Opportunities and Challenges. Sensors.

[14] Microvalve. https://en.wikipedia.org/wiki/Microvalve.

[15] Micropump. https://en.wikipedia.org/wiki/Micropump.

[16] Microfluidics: A General Overview of Microfluidics. https://www.elveflow.com/microfluidic-reviews/general-microfluidics/a-general-overview-of-microfluidics/#:~:text=Microfluidic%20devices%20exploit%20the%20physical,the%20global%20fees%20of%20applications.

[17] Definitions. https://www.ufluidix.com/resources/definitions/.

[18] Scott S.M., Ali Z. (2021). Fabrication Methods for Microfluidic Devices: An Overview. Micromachines.

[19] Nielsen J.B., Hanson R.L., Almughamsi H.M., Pang C., Fish T.R., Woolley A.T. (2020). Microfluidics: Innovations in Materials and Their Fabrication and Functionalization. Anal. Chem..

[20] Raj M.K., Chakraborty S. (2020). PDMS microfluidics: A mini review. J. Appl. Polym. Sci..

[21] Friend J., Yeo L. (2010). Fabrication of microfluidic devices using polydimethylsiloxane. Biomicrofluidics.

[22] Walsh D.I., Kong D.S., Murthy S.K., Carr P.A. (2017). Enabling Microfluidics: From Clean Rooms to Makerspaces. Trends Biotechnol..

[23] Cargou S. Introduction to PDMS Soft-Lithography and Polymer Molding for Microfluidics. https://www.elveflow.com/microfluidic-reviews/soft-lithography-microfabrication/introduction-about-soft-lithography-and-polymer-molding-for-microfluidic/.

[24] Xometry T. Types of 3D Printer Filaments. https://www.xometry.com/resources/3d-printing/types-of-3d-printer-filaments/.

[25] Cheon J., Kim S. (2022). Fabrication and Demonstration of a 3D-printing/PDMS Integrated Microfluidic Device. Recent Prog. Mater..

[26] Cheon J., Kim S. (2019). Intermediate layer-based bonding techniques for polydimethylsiloxane/digital light processing 3D-printed microfluidic devices. J. Micromech. Microeng..

[27] Cargou S. Different Microfluidic Fabrication Techniques. https://www.elveflow.com/microfluidic-reviews/soft-lithography-microfabrication/soft-lithography-fabrication-technics/.

[28] Bedolla M. An Overview of the Photolithography Process. https://www.platypustech.com/an-overview-of-the-photolithography-process.

[29] Photolithography: Patterned Surfaces. https://openwetware.org/wiki/Photolithography:Patterned_Surfaces.

[30] Chin C.D., Linder V., Sia S.K. (2012). Commercialization of microfluidic point-of-care diagnostic devices. Lab Chip.

[31] Kitiara Griffin D.P. (2023). 3D printed microfluidics for bioanalysis: A review of recent advancements and applications. TrAC Trends Anal. Chem..

[32] What Is FDM (Fused Deposition Modeling) 3D Printing?. https://www.hubs.com/knowledge-base/what-is-fdm-3d-printing/.

[33] Ahart M. Types of 3D Printing Technology. https://www.protolabs.com/resources/blog/types-of-3d-printing/.

[34] Prior M. The Different Types of Resins Available for 3D Printing. https://www.3dnatives.com/en/different-types-of-resins-3d-printing-281220225/.

[35] FDM vs. SLA: Compare Filament and Resin 3D Printers. https://formlabs.com/blog/fdm-vs-sla-compare-types-of-3d-printers/.

[36] Guide to Stereolithography (SLA) 3D Printing. https://formlabs.com/blog/ultimate-guide-to-stereolithography-sla-3d-printing/.

[37] Xometry T. All About Digital Light Process (DLP) 3D Printing. https://www.xometry.com/resources/3d-printing/digital-light-process-dlp/.

[38] Nielsen A.V., Beauchamp M.J., Nordin G.P., Woolley A.T. (2020). 3D Printed Microfluidics. Annu. Rev. Anal. Chem..

[39] Sochol R.D., Sweet E., Glick C.C., Wu S.-Y., Yang C., Restaino M., Lin L. (2018). 3D printed microfluidics and microelectronics. Microelectron. Eng..

[40] Naghash T.H., Haghgoo A.M., Bijarchi M.A., Ghassemi M., Shafii M.B. (2024). Performance of microball micromixers using a programmable magnetic system by applying novel movement patterns. Sens. Actuators B Chem..

[41] Kara A., Vassiliadou A., Ongoren B., Keeble W., Hing R., Lalatsa A., Serrano D.R. (2021). Engineering 3D Printed Microfluidic Chips for the Fabrication of Nanomedicines. Pharmaceutics.

[42] Prabhakar P., Sen R.K., Dwivedi N., Khan R., Solanki P.R., Srivastava A.K., Dhand C. (2021). 3D-Printed Microfluidics and Potential Biomedical Applications. Front. Nanotechnol..

[43] Razavi Bazaz S., Rouhi O., Raoufi M.A., Ejeian F., Asadnia M., Jin D., Ebrahimi Warkiani M. (2020). 3D Printing of Inertial Microfluidic Devices. Sci. Rep..

[44] Neuville A., Renaud L., Luu T.T., Minde M.W., Jettestuen E., Vinningland J.L., Hiorth A., Dysthe D.K. (2017). Xurography for microfluidics on a reactive solid. Lab Chip.

[45] Islam M., Natu R., Martinez-Duarte R. (2015). A study on the limits and advantages of using a desktop cutter plotter to fabricate microfluidic networks. Microfluid. Nanofluid..

[46] Laser Cutting—Cutting Processes. https://www.twi-global.com/technical-knowledge/job-knowledge/cutting-processes-laser-cutting-052.aspx.

[47] Gale B., Jafek A., Lambert C., Goenner B., Moghimifam H., Nze U., Kamarapu S. (2018). A Review of Current Methods in Microfluidic Device Fabrication and Future Commercialization Prospects. Inventions.

[48] Khashayar P., Amoabediny G., Larijani B. (2016). Rapid prototyping of microfluidic chips using laser-cut double-sided tape for electrochemical biosensors. J. Braz. Soc. Mech. Sci. Eng..

[49] Nath P., Fung D., Kunde Y.A., Zeytun A., Branch B., Goddard G. (2010). Rapid prototyping of robust and versatile microfluidic components using adhesive transfer tapes. Lab Chip.

[50] Patko D., Mártonfalvi Z., Kovacs B., Vonderviszt F., Kellermayer M., Horvath R. (2014). Microfluidic channels laser-cut in thin double-sided tapes: Cost-effective biocompatible fluidics in minutes from design to final integration with optical biochips. Sens. Actuators B.

[51] Martinez-Lopez J.I., Mojica M., Rodriguez C.A., Siller H.R. (2016). Xurography as a Rapid Fabrication Alternative for Point-of-Care Devices: Assessment of Passive Micromixers. Sensors.

[52] Ma H.L., Urbaczek A.C., Zeferino Ribeiro de Souza F., Augusto Gomes Garrido Carneiro Leao P., Rodrigues Perussi J., Carrilho E. (2021). Rapid Fabrication of Microfluidic Devices for Biological Mimicking: A Survey of Materials and Biocompatibility. Micromachines.

[53] What Is Laser Cutting?—A Definitive Guide to the Process. https://www.twi-global.com/technical-knowledge/faqs/what-is-laser-cutting.

[54] Laser Cutting. https://en.wikipedia.org/wiki/Laser_cutting.

[55] Shahriari S., Patel V., Selvaganapathy P.R. (2023). Xurography as a tool for fabrication of microfluidic devices. J. Micromech. Microeng..

[56] What Are the Methods Used in Microfluidics for Sealing Your Microchips?. https://www.blackholelab-soft-lithography.com/bonding-techniques-in-microfluidics.

[57] Li J., Liang C., Zhang H., Liu C. (2017). Reliable and high quality adhesive bonding for microfluidic devices. MIicro Nano Lett..

[58] Microfluidic Bonding Technology Guide for Polymer Consumables. https://www.flowalliance.com/post/microfluidic-bonding-technology-guide-for-polymer-consumables.

[59] Kiran G., Tsao C. (2022). Recent Advances in Thermoplastic Microfluidic Bonding. Micromachines.

[60] Trinh K.T.L., Thai D.A., Lee N.Y. (2022). Bonding Strategies for Thermoplastics Applicable for Bioanalysis and Diagnostics. Micromachines.

[61] Sypabekova M., Hagemann A., Kleiss J., Morlan C., Kim S. (2023). Optimizing an Optical Cavity-Based Biosensor for Enhanced Sensitivity. IEEE Sens. J..

[62] Wan A.M., Moore T.A., Young E.W. (2017). Solvent Bonding for Fabrication of PMMA and COP Microfluidic Devices. J. Visualized Exp..

[63] Michael C. Biocompatible Bonding of Microfluidic Consumables. https://www.google.com/url?sa=t&rct=j&q=&esrc=s&source=web&cd=&ved=2ahUKEwjnm6q3k4GGAxUq5ckDHYuMDZAQFnoECBYQAQ&url=https%3A%2F%2Fwww.micronit.com%2Fdam%2Fjcr%3Aef1d6e73-0022-45e5-89ab-c953f9944bd9%2FWhitepaper%2520Biocompatible%2520bonding.pdf&usg=AOvVaw3so5-RW0JFbb3f9XNhYgDm&opi=89978449.

[64] Shu Pei Ng F.E.W., Tay N.B. (2016). Low Distortion Solvent Bonding of Microfluidic Chips. Procedia Eng..

[65] Matsuura Y., Takehira M., Joti Y., Ogasahara K., Tanaka T., Ono N., Kunishima N., Yutani K. (2015). Thermodynamics of protein denaturation at temperatures over 100 °C: CutA1 mutant proteins substituted with hydrophobic and charged residues. Sci. Rep..

[66] Thompson C.S., Abate A.R. (2013). Adhesive-based bonding technique for PDMS microfluidic devices. Lab Chip.

[67] Serra M., Pereiro I., Yamada A., Viovy J.L., Descroix S., Ferraro D. (2017). A simple and low-cost chip bonding solution for high pressure, high temperature and biological applications. Lab Chip.

[68] Dabaghi M., Tiessen N., Cao Q., Chandiramohan A., Saraei N., Kim Y., Gupta T., Selvaganapathy P.R., Hirota J.A. (2021). Adhesive-Based Fabrication Technique for Culture of Lung Airway Epithelial Cells with Applications in Cell Patterning and Microfluidics. ACS Biomater. Sci. Eng..

[69] The Benefits of Tape in Microfluidic Devices: Tape’s New “Role” in Health Care. https://www.adhesiveapps.com/tape-in-microfluidic-devices/.

[70] Fan Y., Liu S., He J., Gao K., Zhang Y. (2017). Rapid prototyping of flexible multilayer microfluidic devices using polyester sealing film. Microsyst. Technol..

[71] Jang I., Carrao D.B., Menger R.F., Moraes de Oliveira A.R., Henry C.S. (2020). Pump-Free Microfluidic Rapid Mixer Combined with a Paper-Based Channel. ACS Sens..

[72] Carrell C., Kava A., Nguyen M., Menger R., Munshi Z., Call Z., Nussbaum M., Henry C. (2019). Beyond the lateral flow assay: A review of paper-based microfluidics. Microelectron. Eng..

[73] Martinez A.W., Phillips S.T., Whitesides G.M. (2008). Three-dimensional microfluidic devices fabricated in layered paper and tape. Proc. Natl. Acad. Sci. USA.

[74] Martinez A.W., Phillips S.T., Nie Z., Cheng C.M., Carrilho E., Wiley B.J., Whitesides G.M. (2010). Programmable diagnostic devices made from paper and tape. Lab Chip.

[75] Barrios C.A. (2020). Pressure Sensitive Adhesive Tape: A Versatile Material Platform for Optical Sensors. Sensors.

[76] Panraksa Y., Jang I., Carrell C.S., Amin A.G., Chailapakul O., Chatterjee D., Henry C.S. (2022). Simple manipulation of enzyme-linked immunosorbent assay (ELISA) using an automated microfluidic interface. Anal. Methods.

[77] Rivet C., Lee H., Hirsch A., Hamilton S., Lu H. (2011). Microfluidics for medical diagnostics and biosensors. Chem. Eng. Sci..

[78] Kratz S.R.A., Eilenberger C., Schuller P., Bachmann B., Spitz S., Ertl P., Rothbauer M. (2019). Characterization of four functional biocompatible pressure-sensitive adhesives for rapid prototyping of cell-based lab-on-a-chip and organ-on-a-chip systems. Sci. Rep..

[79] 3M Thin Bonding: Double Coated & Adhesive Transfer Tapes. https://www.3m.com/3M/en_US/bonding-and-assembly-us/double-sided-tape/thin-bonding-tape/.

[80] Fleenor S. Adhesive Tape Types: 5 Constructions. https://www.walkertapeconverting.com/adhesive-tape-types-5-constructions/.

[81] Lucas K., Oh J., Hoelzl J., Weissleder R. (2022). Cellular point-of-care diagnostics using an inexpensive layer-stack microfluidic device. Lab Chip.

[82] Chambers S. The Basics of Pressure Sensitive Adhesive Tape. https://www.strouse.com/blog/the-basics-of-pressure-sensitive-adhesive-tape.

[83] Ira S. Rubber vs. Acrylic Based Adhesives. https://www.hookandloop.com/blog/rubber-vs-acrylic-based-adhesives.

[84] Silicone vs. Acrylic Adhesive—A Comparison. http://www.industrialrubbergoods.com/articles/silicone-acrylic-adhesive.html.

[85] Acrylic PMMA Chemical Compatiblity Chart. https://www.industrialspec.com/resources/acrylic-aka-pmma-chemical-compatiblity-chart/.

[86] Chemical Compatibility Chart. https://jehbco.com.au/products/chemical-compatibility-chart/.

[87] Yuen P.K., Goral V.N. (2010). Low-cost rapid prototyping of flexible microfluidic devices using a desktop digital craft cutter. Lab Chip.

[88] Alvarez-Braña Y., Etxebarria-Elezgarai J., Ruiz de Larrinaga-Vicente L., Benito-Lopez F., Basabe-Desmonts L. (2021). Modular micropumps fabricated by 3D printed technologies for polymeric microfluidic device applications. Sens. Actuators B.

[89] Etxebarria-Elezgarai J., Alvarez-Braña Y., Garoz-Sanchez R., Benito-Lopez F., Basabe-Desmonts L. (2020). Large-Volume Self-Powered Disposable Microfluidics by the Integration of Modular Polymer Micropumps with Plastic Microfluidic Cartridges. Ind. Eng. Chem. Res..

[90] Hassanpour-Tamrin S., Sanati-Nezhad A., Sen A. (2021). A simple and low-cost approach for irreversible bonding of polymethylmethacrylate and polydimethylsiloxane at room temperature for high-pressure hybrid microfluidics. Sci. Rep..

[91] Buzzin A., Iannascoli L., Muzi M., Veroli A., Caputo D., de Cesare G., Maiolo L., Maita F., Ricci G. Integrated 3D Microfluidic Device for Impedance Spectroscopy in Lab-on-Chip Systems. Proceedings of the 2019 IEEE 8th International Workshop on Advances in Sensors and Interfaces.

[92] Nascetti A., Mirasoli M., Marchegiani E., Zangheri M., Costantini F., Porchetta A., Iannascoli L., Lovecchio N., Caputo D., de Cesare G. (2019). Integrated chemiluminescence-based lab-on-chip for detection of life markers in extraterrestrial environments. Biosens. Bioelectron..

[93] Stallcop L.E., Alvarez-Garcia Y.R., Reyes-Ramos A.M., Ramos-Cruz K.P., Morgan M.M., Shi Y., Li L., Beebe D.J., Domenech M., Warrick J.W. (2018). Razor-printed sticker microdevices for cell-based applications. Lab Chip.

[94] Sui C., Zilberberg J., Lee W. (2022). Microfluidic device engineered to study the trafficking of multiple myeloma cancer cells through the sinusoidal niche of bone marrow. Sci. Rep..

[95] Wang Y., Seidel M. (2021). Strategy for fast manufacturing of 3D hydrodynamic focusing multilayer microfluidic chips and its application for flow-based synthesis of gold nanoparticles. Microfluid. Nanofluid..

[96] Vasconez Martinez M.G., Reihs E.I., Stuetz H.M., Hafner A., Brandauer K., Selinger F., Schuller P., Bastus N., Puntes V., Frank J. (2024). Using Rapid Prototyping to Develop a Cell-Based Platform with Electrical Impedance Sensor Membranes for In Vitro RPMI2650 Nasal Nanotoxicology Monitoring. Biosensors.

[97] Langer K., Joensson H.N. (2020). Rapid Production and Recovery of Cell Spheroids by Automated Droplet Microfluidics. SLAS Technol..

[98] Naik A.R., Zhou Y., Dey A.A., Arellano D.L.G., Okoroanyanwu U., Secor E.B., Hersam M.C., Morse J., Rothstein J.P., Carter K.R. (2021). Printed microfluidic sweat sensing platform for cortisol and glucose detection. Lab Chip.

[99] Shahriari S., Damodara S., Selvaganapathy P.R. (2024). Isoelectric trapping and discrimination of histones from plasma in a microfluidic device using dehydrated isoelectric gate. Microchim Acta.

[100] Zamora V., Marx S., Arndt-Staufenbiel N., Janeczka C., Havlik G., Queisser M., Schröder H. Laser-Microstructured Double-Sided Adhesive Tapes for Integration of a Disposable Biochip. Proceedings of the Eurosensors 2017.

[101] Dossi N., Petrazzi S., Terzi F., Toniolo R., Bontempelli G. (2019). Electroanalytical cells pencil drawn on PVC supports and their use for the detection in flexible microfluidic devices. Talanta.

[102] Gerber L.C., Kim H., Riedel-Kruse I.H. (2015). Microfluidic assembly kit based on laser-cut building blocks for education and fast prototyping. Biomicrofluidics.

[103] Lin H., Zhao Y., Lin S., Wang B., Yeung C., Cheng X., Wang Z., Cai T., Yu W., King K. (2019). A rapid and low-cost fabrication and integration scheme to render 3D microfluidic architectures for wearable biofluid sampling, manipulation, and sensing. Lab Chip.

[104] Hwang J., Kim S., Kim Y., Song H., Park C., Kim J. (2015). Implementation of PCB-Based PCR Chip Using Double-Sided Tape. Int. J. Control Autom..

[105] Belova A.M., Basmanov D.V., Prusakov K.A., Lazarev V.N., Klinov D.V. (2019). A Microfluidic Platform for the Development of a Biosensor Based on Genetically Modified Helicobacter pylori Single Cells. Biophysics.

[106] Carter S.D., Barbe L., Tenje M., Mestres G. (2020). Exploring microfluidics as a tool to evaluate the biological properties of a titanium alloy under dynamic conditions. Biomater. Sci..

[107] Dutta G., Regoutz A., Moschou D. (2020). Enzyme-assisted glucose quantification for a painless Lab-on-PCB patch implementation. Biosens. Bioelectron..

[108] Grant N., Geiss B., Field S., Demann A., Chen T.W. (2021). Design of a Hand-Held and Battery-Operated Digital Microfluidic Device Using EWOD for Lab-on-a-Chip Applications. Micromachines.

[109] Guner H., Ozgur E., Kokturk G., Celik M., Esen E., Topal A.E., Ayas S., Uludag Y., Elbuken C., Dana A. (2017). A smartphone based surface plasmon resonance imaging (SPRi) platform for on-site biodetection. Sens. Actuators B.

[110] Ribeiro M., Ali P., Metcalfe B., Moschou D., Rocha P.R.F. (2021). Microfluidics Integration into Low-Noise Multi-Electrode Arrays. Micromachines.

[111] Podunavac I., Hinić S., Kojić S., Jelenčiakova N., Radonić V., Petrović B., Stojanović G. (2021). Microfluidic Approach for Measurements of pH, O2, and CO2 in Saliva. Sens. Mater..

[112] Lee J.H., Van der Linden C., Diaz F.J., Wong P.K. (2022). A reconfigurable microfluidic building block platform for high-throughput nonhormonal contraceptive screening. Lab Chip.

[113] Shin J., Kasama T., Miyake R. (2022). Development of cellulosic material-based microchannel device capable of fluorescence immunoassay of microsamples. Anal. Bioanal. Chem..

[114] Delgado P., Luna C.A., Dissanayaka A., Oshinowo O., Waggoner J.J., Schley S., Fernadez T., Myers D.R. (2022). An economical in-class sticker microfluidic activity develops student expertise in microscale physics and device manufactoring. arXiv.

[115] Ragavendar M.S., Jayaraman S., Ramya V.M., Roy R., Manwani H. (2014). Performance evaluation of low cost microfluidic chips made using a digital craft cutter for point of care applications in nucleic acid tests. Annu. Int. Conf. IEEE Eng. Med. Biol. Soc..

[116] Lopez-Munoz G.A., Estevez M.C., Pelaez-Gutierrez E.C., Homs-Corbera A., Garcia-Hernandez M.C., Imbaud J.I., Lechuga L.M. (2017). A label-free nanostructured plasmonic biosensor based on Blu-ray discs with integrated microfluidics for sensitive biodetection. Biosens. Bioelectron..

[117] Singer R., Hirota J.A., Dabaghi M. (2023). Irreversible PDMS bonding using flame activation of adhesives for fabrication of microfluidic and organ-on-chip devices. Mater. Lett..

[118] Vila J.C., Castro-Aguirre N., Lopez-Munoz G.A., Ferret-Minana A., De Chiara F., Ramon-Azcon J. (2021). Disposable Polymeric Nanostructured Plasmonic Biosensors for Cell Culture Adhesion Monitoring. Front. Bioeng. Biotechnol..

[119] Channon R.B., Menger R.F., Wang W., Carrão D.B., Vallabhuneni S., Kota A.K., Henry C.S. (2021). Design and application of a self-pumping microfluidic staggered herringbone mixer. Microfluid. Nanofluid..

[120] Akuoko Y., Hanson R.L., Harris D.H., Nielsen J.B., Lazalde E., Woolley A.T. (2021). Rapid and simple pressure-sensitive adhesive microdevice fabrication for sequence-specific capture and fluorescence detection of sepsis-related bacterial plasmid gene sequences. Anal. Bioanal. Chem..

[121] Fleenor S. Low Temperature Double-Sided Tape. https://www.walkertapeconverting.com/low-temperature-double-sided-tape/.

[122] The Effects of Low Temperatures on Pressure-Sensitive Adhesives (PSAs). https://www.budnick.com/learning-center/blog/the-effects-of-low-temperatures-on-pressure-sensitive-adhesives-psas.

[123] Achieving the Best Bond. https://www.can-dotape.com/adhesive-tape-consultant/achieving-the-optimum-bond/.

[124] Spence J.S. (2018). All About Lasers. Engrav. J..

